# Alignment-free Transcriptomic and Metatranscriptomic Comparison Using Sequencing Signatures with Variable Length Markov Chains

**DOI:** 10.1038/srep37243

**Published:** 2016-11-23

**Authors:** Weinan Liao, Jie Ren, Kun Wang, Shun Wang, Feng Zeng, Ying Wang, Fengzhu Sun

**Affiliations:** 1 Department of Automation, Xiamen University, Xiamen, Fujian, 361005 China; 2 Molecular and Computational Biology Program, University of Southern California, Los Angeles, California, CA 90089 USA; 3 Center for Computational Systems Biology, Fudan University, Shanghai 200433, China

## Abstract

The comparison between microbial sequencing data is critical to understand the dynamics of microbial communities. The alignment-based tools analyzing metagenomic datasets require reference sequences and read alignments. The available alignment-free dissimilarity approaches model the background sequences with Fixed Order Markov Chain (FOMC) yielding promising results for the comparison of microbial communities. However, in FOMC, the number of parameters grows exponentially with the increase of the order of Markov Chain (MC). Under a fixed high order of MC, the parameters might not be accurately estimated owing to the limitation of sequencing depth. In our study, we investigate an alternative to FOMC to model background sequences with the data-driven Variable Length Markov Chain (VLMC) in metatranscriptomic data. The VLMC originally designed for long sequences was extended to apply to high-throughput sequencing reads and the strategies to estimate the corresponding parameters were developed. The flexible number of parameters in VLMC avoids estimating the vast number of parameters of high-order MC under limited sequencing depth. Different from the manual selection in FOMC, VLMC determines the MC order adaptively. Several beta diversity measures based on VLMC were applied to compare the bacterial RNA-Seq and metatranscriptomic datasets. Experiments show that VLMC outperforms FOMC to model the background sequences in transcriptomic and metatranscriptomic samples. A software pipeline is available at https://d2vlmc.codeplex.com.

Understanding the factors affecting microbe composition and the relationship between microbes and hosts depends on accurate comparison of microbial communities^1^. The high-throughput sequencing data of microbial communities harbor the whole DNA/RNA information for elaborate and comprehensive comparison. Generally, alignment-based sequencing comparison methods, such as the Smith-Waterman algorithm^2^ and BLAST^3^, have been extensively used to compare microbial communities based on short read data. The reads are usually mapped to known genome or pathway databases, followed by estimation of the abundance levels of genomes and/or gene families. Microbial communities are then compared based on the abundance levels. Recently, several computational tools including Kraken^4^, Clark^5^ and Kaiju^6^, have been developed for fast taxonomic classification of sequencing reads using hash-based k-mer indices built from reference sequences. These methods achieved comparable accuracy as that of the traditional BLAST programs, yet they are up to ~900 times^4^ faster than Megablast and ~10 times^4^ faster than MetaPhlan^7^. In addition, MetaPhlan^7^ uses only known marker genes. If communities do not share any marker genes included in MetaPhlan, the program will not be able to report the relationships among the communities. On the other hand, Kraken^4^, Clark^5^ and Kaiju^6^ do not have such limitations. However, the reference-based comparison approaches have several limitations: (1) Dependency on sequences of reference genomes or genes. However, a large amount of microbial genomes and gene families are unknown or incomplete, which affects the accuracy and completeness of the analysis. According to current publications, for metatranscriptomic data, there were about 19–42% unassigned reads in marine water samples^8^, about 10–20% unassigned reads in human small intestine microbiota^9^, and up-to 50% reads that cannot be assigned to reference databases in oceans with large phytoplankton^10^. Therefore, alignment-based methods are not applicable for microbial communities with a large amount of dark matters. (2) Current tools analyzing the microbial communities were mostly designed for metagenomics based on mark genes, such as 16S rRNA. However, for the metatranscriptomic dataset, ribosomal RNA (rRNA) transcripts are often required to be depleted in order to maximize mRNA recovery^8^,^9^. Therefore, the metagenomic tools based on 16S rRNA marker genes are not suitable to analyze metatranscriptomic data. Among the limited metatranscriptomic analytic tools, some were designed for Illumina paired-end^10^ or single/paired-end data^11^, or only used to evaluate the gene expression level^12^. A previous study^11^ compared four taxonomical classification tools based on a common metatranscriptomic data and obvious differences among the taxonomical analytic results were observed, which was the second figure in original paper^11^. (3) Sequence assembly is time-consuming and challenging especially for metagenome/metatranscriptome when organisms share a high volume of homologous sequences. Different assembled contigs were obtained for the same reads when using different assembly tools. Therefore, alignment-free methods provide a promising alternative for microbial community comparison, eliminating the requirements of reference sequences and assembly.

One type of alignment-free methods is based on the frequencies of *k-*tuples (*k-*words, *k*-mers or *k-*grams)^13^. A *k-*tuple is a contiguous sequence of length *k*. Previous studies indicate that relative *k-*tuple frequencies are similar across different regions of the same genome, but differ between genomes^14^. One of the earliest similarity measures between two sequences is *D*_2_ which measures the total number of matched *k*-tuples between two long sequences^13^. However, theoretical studies have shown that the distribution of *D*_2_ is dominated by the variance in the number of occurrences of *k*-tuples along individual sequences and less by the relationship between sequences^15^. Consequently, other similarity measures have been developed with different normalization, centralization and background models in an attempt to modify *D*_2_, including 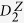
16, 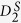
15, 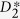
17, 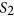
18,19 and CVTree measures. Subsequently, normalized dissimilarity measures^20^ based on *D*_2_,
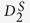
and 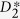, including 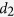, 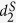 and 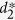
1,21 with range between 0 and 1, were developed for high-throughput sequencing data. Indeed, previous studies^22^ showed that *k-*tuple-based dissimilarity measures are effective in revealing group relationships and gradient relationships among metagenomic and metatranscriptomic samples and that 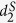 and 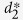 achieved the best performances in most comparisons of microbial communities.

However, the utility of 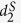
and 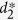 depends on a proper probability model for background genomes. To address this gap, Fixed Order Markov Chains (FOMC) were used to model the background genome sequences, as reported in previous studies^22^,^23^. There are several limitations during the applications of FOMC: (1) The order of Markov Chain (MC) needs to be set manually. However, for most microbial communities, there is no prior knowledge available for setting the MC order. (2) Furthermore, it is hard to model probabilities of different tuples using a single fixed order MC, and FOMC is not structurally rich. There are *n*^*r*^ × (n − 1) independent parameters for an *r*-th order MC, where *n* is the number of states, that is, *n* = 4 for DNA or RNA sequences. When the order *r* equals 2 or 3, the number of parameters for the model is 48 or 192, respectively. There are no FOMCs with number of parameters between 48 and 192. (3) Thus, the number of parameters grows exponentially with the increase of order *r.* When sequencing depth is relatively low, the parameters, with their number growing exponentially with the increase of MC order in FOMC models, cannot be accurately estimated.

With this in mind, we introduced Variable Length Markov Chains^24^ (VLMC) as an alternative for FOMC to model the background genomes of microbial community in this study. VLMC adaptively determines the order of MC based on the sequence data, thus eliminating manual selection. Additionally, the number of variables in VLMC is flexible. VLMC was originally designed for modeling one long sequence and was represented as a context tree structure^24^,^25^. For high-throughput sequencing of short reads, the likelihood of underlying, or unobserved sequences cannot be calculated. As a result, the rules for pruning the tree are not clearly defined. Therefore, we first developed strategies to determine the parameters for building a context tree and then extended VLMC for high-throughput sequencing of short reads. Thus, the complete context tree is constructed from these short reads, which typically overfits the data. The number of independent parameters is Num(nodes) × 3, where the Num(nodes) is the total number of nodes in the context tree except the root-node. The tree is then pruned according to a local decision rule. Using VLMC for background modeling, 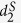 and 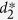 measures were then applied to compare transcriptomic or metatranscriptomic datasets. From the obtained dissimilarities among samples, the clustering trees were evaluated based on the triples distance^26^ between the reference and resulting trees. Experimental results show that VLMC models the position dependency in the nucleotide sequences better than FOMC, and since it is free from order selection required by FOMC, VLMC is easier to apply. Our studies also show that VLMC probability models combined with 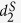 and 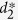 measures exhibit superior performance in clustering metatranscriptomic samples when compared to previous approaches.

## Results

### Design of experiments

In order to explore the performance of 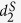 and 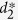 with VLMC, we designed experiments with one simulated dataset and four real datasets. The simulated metatranscriptomic dataset is composed of 90 samples belonging to 3 different groups with 5,000 genes from 5 microbes. Real dataset 1 consists of 18 and 22 RNA-Seq datasets from marine microbial eukaryotes. For 18 RNA-Seq datasets, the molecular phylogeny^27^ was reconstructed based on the 18S rRNA genes with maximum likelihood (ML) method. The ML phylogenetic tree was then used to evaluate the ability of VLMC as background model, combined with 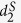 and 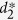 measures to compare their relationships based on the high-throughput sequencing data of individual species. For 22 RNA-Seq datasets, phylogenetic tree was built with Bayesian inference using MrBayes^28^ program. Dataset 2 contains 88 metatranscriptomic samples collected from the Global Ocean Sampling Expedition (GOSE), and they were used to study the effect of VLMC-based measures in identifying group relationships. Dataset 3 consists of 8 metagenomic and 8 metatranscriptomic samples from ocean depths of 25 m, 75 m, 125 m and 500 m. Dataset 4 consists of 14 metatranscriptomic samples from depths of 0.03 m and 0.08 m within a typical iron-rich microbial mat. Datasets 3 and 4 were used to study the performance of VLMC-based measures in revealing environmental gradient relationships. The triples distances were applied to evaluate the consistency between the reference and clustering trees from alignment-free measures.

There is no rigorous criterion to decide the optimal length *k* for *k*-tuples. However, according to our previous experiments, generally the optimal *k* is 6–9. For comparison, 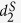 and 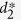 with *0–4th* order FOMC, three *L*_*p*_*-norm* measures and *d*_2_ were also applied.

### Experiment 1: Detecting group relationships among simulated metatranscriptomic datasets

Using a similar simulation strategy as developed in Martinez *et al.*^11^, we simulated three groups of synthetic mock communities with different expression levels using Polyester^12^, an RNA-Seq simulation tool. Five most abundant microbial genomes in human gut were selected based on Qin *et al.*^29^: *Bacteroides vulgatus ATCC 8482*, *Ruminococcus torques L2−14*, *Faecalibacterium prausnitzii SL3/3*, *Bacteroides thetaiotaomicron VPI-5482* and *Parabacteroides distasonis ATCC 8503*. For each bacterium, a subsample of 1000 genes was randomly selected without replacement. Based on the mock community consisting of 5,000 genes from the five bacteria, we set three group centers with different gene expression levels as follows:

1. Among the 5000 genes, 20% showed 4-fold overexpression, 20% showed 4-fold under-expression, and 60% were normally-expressed. The simulation tool Polyester^12^ uses a fold change vector to specify the different expression levels among transcripts. Polyester generates the baseline read numbers from a negative binomial distribution with a preset mean value (default mean = 300), and then multiply the baseline numbers by the fold changes to simulate the transcripts with different expression levels. As shown in equation (1), *A* is the basic fold change vector, and 20% of the elements equal 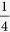, 20% equal 4, and the others equal 1.

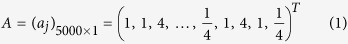


We then generated 90 samples belonging to 3 groups each containing 30 samples using the simulation strategy as in Jiang *et al.*^22^, shown in steps (2) and (3).

(2)The three group centers *A*_1_ *A*_2_ and *A*_3_ were generated as equation (2). *Norm(μ*,*σ*^2^) indicates the normal distribution with mean *μ* and variance *σ*^2^.


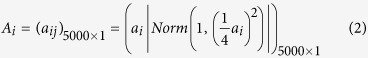


(3) For the *q*^*th*^ sample 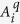 within group *A_i_*, the expression level vector 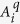 were generated using equation (3).


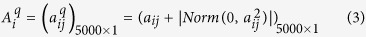


Based on the generated 90 expression level vectors, 90 metatranscriptomic sequencing data were simulated and the read length was 76 bp.

**Figure 1:**
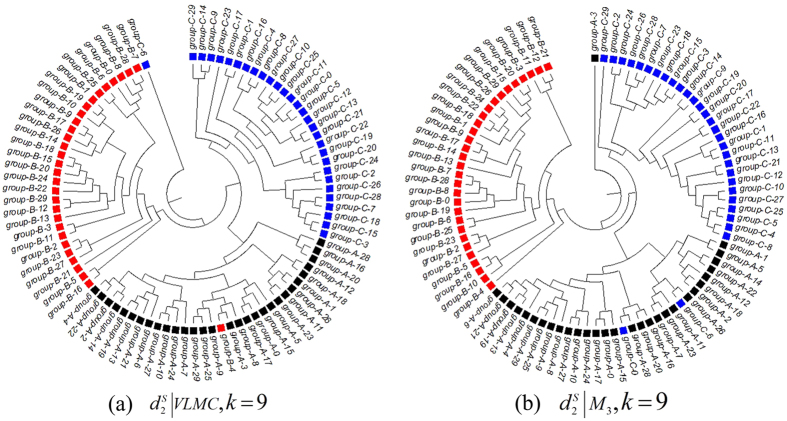
The clustering trees based on different models for the 90 simulation samples in Experiment 1. (**a**) The best clustering tree on VLMC. (**b**) The best clustering tree when using FOMC and *l_p_-norm* measures. *Samples are divided into three groups A–C. Each group has 30 samples numbered from 0 to 29.

The best hierarchical clustering trees with VLMC and FOMC are shown in Fig. 1, and the corresponding triples distances are shown in Table 1. Clear groups of three simulated datasets among samples can be observed for both VLMC and FOMC. The best clustering trees with the smallest triples distance for VLMC and FOMC are both obtained in *k* = 9 and using 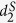 dissimilarity measure. From the clustering tree in Fig. 1, it is clear that the tree built based on VLMC is more similar to the true tree than that build based on FOMC. Quantitatively, the smallest triples distance for VLMC and FOMC are 42,973 and 43,043, respectively, where VLMC outperforms FOMC with less misclassification.

### Experiment 2: Comparison based on RNA-Seq data of Marine Microbial Eukaryotes

**Figure 2:**
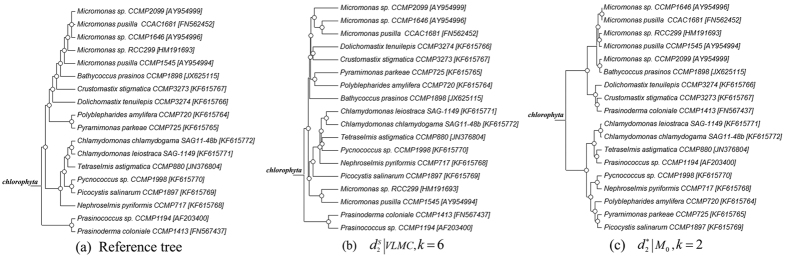
The reference tree and the best clustering trees based on VLMC and FOMC models for 18 RNA-Seq data in Experiment 2. (**a**) The molecular phylogenic tree of the 18 RNA-seq built with Maximum likelihood method on the 18S rRNA genes. (**b**) The best clustering tree with VLMC. (**c**) The best clustering tree when using FOMC and *L_p_-norm* measures. *Samples are labeled as the Organisms-Strain-18S rRNA. For example, *Micromonas pusilla CCAC1681* [FN562452] represents the organism *Micromonas pusilla* from strain *CCAC1681* and the 18S rRNA used to construct this ML tree is *FN562452*. Details about sample labels can be found in Supplementary Table S4 in section 2.

RNA-Seq data of 18 marine eukaryotes were downloaded from “The Marine Microbial Eukaryote Transcriptome Sequencing Project”^30^. The 18 eukaryotes are from the Phylum *Chlorophyta,* and the sample information is listed in Table S1 in Supplementary Section 1.1. The reference tree of the eukaryotes was extracted from the molecular phylogenetic tree built from a previous study^27^ that reconstructed the tree by maximum likelihood (ML) based on the 18S rRNA gene from a genome sequence or RNA-seq-based transcriptome assembly, shown in Supplementary Figure 1 of their paper^27^. Figure 2a shows the resulting ML of the 18 eukaryotes and it is used as a reference tree in our study. The bootstrap supports for the nodes in the phylogenetic reference tree are higher than 65%. The bootstrap support values of the nodes were calculated based on 1,000 replicates of the data with the same substitution model. The Bayesian posterior probabilities of the nodes in the tree were higher than 90%. The Bayesian analyses were performed with two independent runs with 1,000,000 generations per run. After a burn-in of 350,000 trees (that were discarded) per run, the remaining trees were used to reconstruct a consensus tree and to obtain posterior probabilities for node supports^27^.

Table 2 shows the triples distance between the reference and clustering trees using various dissimilarity measures and tuple length. The best clustering result with the smallest triples distance of 177 is obtained by VLMC using the dissimilarity measure 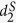 and tuple length *k* = 6, as shown in Fig. 2b. The topological structure is similar to that of the reference phylogenetic tree which basically includes three groups. The smallest triples distance for FOMC is 318, which was achieved by using 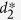 with 0-order MC and *k* = 2, as shown in Fig. 2c. Its overall topological structure of the clustering results is different with the phylogenetic tree in Fig. 2a. The clustering result based on VLMC is obviously better than the result based on the FOMC model.

We also analyzed RNA-Seq data from another set consisting of 22 Marine Microbial Eukaryotes from the Phylum *Bacillariophyta*, *Chlorophyta,* and *Cryptophyta.* The phylogenetic tree was built using MrBayes^28^ based on multiple alignments of 18S rRNA sequences using the default settings, and it was used as a reference tree for evaluations. The score for each branch is the Bayesian posterior probability of each partition or clade in the tree. It is the fraction of times that the partition or clade appears in the set of sampled posterior trees. The total number of samples generated from the posterior probability distribution is 1,000,000, and the beginning 25% of the samples were treated as burn-in and were discarded. The three groups *Ch*, *Cr* and *Ba* were clearly clustered to different groups with 100% posterior probabilities. Two internal branches in group *Ba* have Bayesian posterior probabilities less than 100%. The corresponding results for clustering the 22 species based on transcriptome data using FOMC and VLMC were shown in Supplementary Section 1.2. The experiment also shows the superior performance of VLMC over FOMC.

### Experiment 3: Comparison based on 88 global ocean metatranscriptomic samples

**Figure 3:**
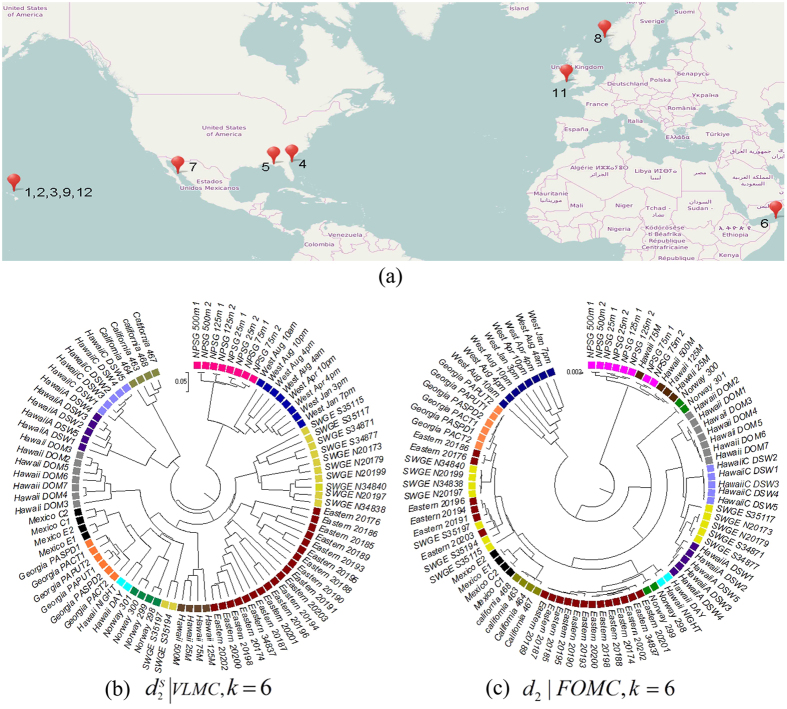
Locations of the 88 metatranscriptomic samples from global ocean, the reference tree, and the clustering trees based on different dissimilarity measures and background sequence models in Experiment 3. (**a**) The distribution of the collecting locations. The map is based on OpenStreetMap and the cartography in the OpenStreetMap map tiles is licensed under CCBY-SA (www.openstreetmap.org/copyright). The license terms can be found on the link: http://creativecommons.org/licenses/by-sa/2.0/. The location labels are marked with the coordinates of sample-collecting locations in Supplementary Table S4 in section 2. (**b**) The clustering tree with VLMC using 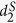 and *k* = 6. (**c**) The clustering tree with FOMC using 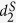 and *k* = 6. *‘SWGE’ (Dataset 10 in Supplemental Table S4 in section 2) samples were collected from different locations with two research cruises in the Equatorial North Atlantic Ocean and South Pacific Subtropical gyre.

In this experiment, 88 metatranscriptomic samples collected from different global ocean locations were analyzed. These samples were downloaded from 12 different projects from Microbe (http://data.imicrobe.us/, originally belonging to CAMERA) and NCBI with 454 pyrosequencing. The descriptions and dataset IDs are given in Table S4 in Supplementary Section 2. Figure 3a shows the locations of these 88 samples. Twenty-three samples are from the subtropical north Pacific (Hawaiian), 4 from the Mexican Gulf, 4 from the California Gulf, 4 from the Norwegian Fjord, 6 from Sapelo Island (Georgia), 8 from the North Atlantic Ocean (West English Channel), 8 from North Pacific Subtropical Gyre (NPSG), and 19 from Eastern Equatorial Atlantic Ocean mixed with Amazon River plume. In addition, 12 samples were collected from different locations of Equatorial North Atlantic Ocean and South Pacific Subtropical Gyre. The map for the distribution of collecting locations was based on OpenStreetMap, and the cartography in the OpenStreetMap map tiles is licensed under CCBY-SA (www.openstreetmap.org/copyright). The license terms can be found on the link: http://creativecommons.org/licenses/by-sa/2.0/.

The clustering trees with 6-tuples based on 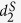 using VLMC and *d*_2_ using FOMC are shown in Fig. 3c. In VLMC, clear groups of different locations among samples can be seen. Except for the two samples from “SWGE”, all other samples are consistently grouped with the marine locations. The communities with proximate latitudes, including Eastern Equa, Atlan_Amazon and SWGE, are clustered first, which is consistent with our understanding that these communities should have greater similarity of gene expression profiles. For FOMC, samples from SWGE and the Amazon River are both scattered into several parts of the clustering. VLMC-based measures reveal location relationships of these 88 global ocean metatranscriptomic samples.

### Experiment 4: Comparison of gradient relationship based on metatranscriptomic samples from different ocean depths

The gene expression profile of microbes can be affected by environmental factors, such as ocean depth, temperature, or pH. To evaluate the performance of the different dissimilarity measures and background sequence models in recovering the gradient relationships of microbial communities, we studied 8 metagenomic and 8 metatranscriptomic samples from depths of 25 m, 75 m, 125 m and 500 m (two replicate samples for each depth) of North Pacific Subtropical Gyre (NPSG) in ALOHA stations^31^ (dataset 12 in Table S4 in Supplementary Section 2).

**Figure 4:**
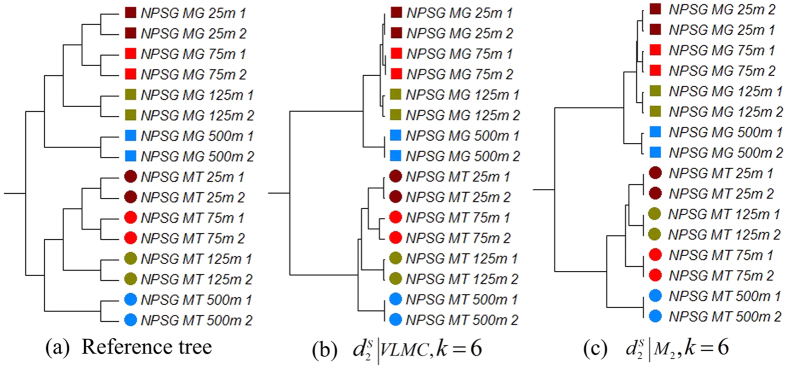
The reference and clustering trees based on various models for different depths of metatranscriptomic marine samples in Experiment 4. (**a**) Reference tree of different depths of metatranscriptomic samples from the ocean. (**b**) The best clustering tree with VLMC background sequence model. (**c**) The best clustering tree when using FOMC and *l_p_-norm* measures.

Table 3 shows the triples distance between the reference tree and the derived clustering trees using different dissimilarity measures and background sequence models. Using the VLMC background sequence model, both 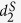 and 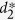 can recover the reference tree. The best results from both VLMC and FOMC background sequence models show clear separations between metagenomic and metatranscriptomic groups, as shown in Fig. 4b,c, respectively. For both background sequence models, samples from the same depth are clustered first, then the samples belonging to the photic zone (25 m, 75 m and 125 m) are merged, and, finally, samples belonging to the mesopelagic zone (500 m). However, for the FOMC background sequence model, the metatranscriptomic samples from 25 m and 125 m are clustered first, which is inconsistent with gradient relationships. In contrast, VLMC background sequence model produces clustering of metagenomic and metatranscriptomic samples as expected with 25 and 50 m first and then 125 m.

### Experiment 5: Comparison of gradient relationships based on metatranscriptomic samples from different iron-rich microbial mats

**Figure 5:**
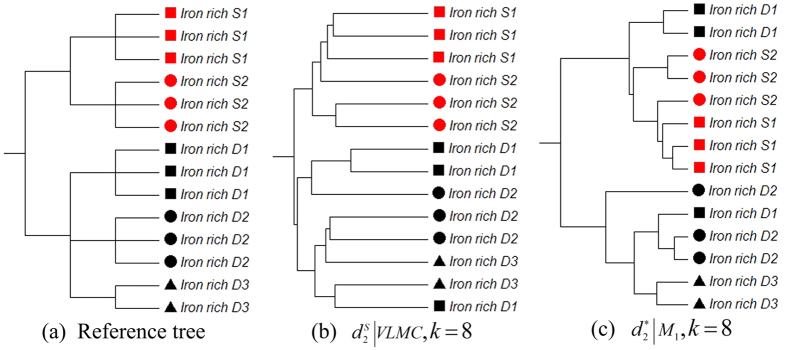
Reference and clustering trees based on different background sequence models for the metatranscriptomic mat samples in Experiment 5. (**a**) Reference tree of the microbial mat data in experiment 5. (**b**) The best clustering tree with the VLMC background sequence model. (**c**) The best clustering tree with the FOMC background sequence model and *l_p_*-*norm* measures.

A microbial mat is a multilayered sheet of microorganisms, mainly bacteria and archaea. Previous studies^32^ found clear phylogenetic stratification between the surface and the deeper regions of the microbial mat where iron-oxidizing bacteria dominated the community in the upper layers, and methanothrophs contributed to the majority of sequences in the deeper layers. Therefore, in this experiment, we used our methods to study 14 metatranscriptomic samples^32^ to evaluate gradient relationships at different depths of the microbial mat. As shown in Figure S2 in Supplementary Section 3, the sampling site is a slow-flowing stream where two collection sites (S1, S2) are placed at 1 cm in depth (surface water), and three collection sites (D1, D2.D3) are placed in deeper regions of 7–9 cm. Three samples were collected at every collection site, except D3, where only two samples were harvested. The descriptions and dataset IDs of these samples can be found in Table S5 in Supplementary Section 3. Figure 5a shows the reference tree of the 14 microbial mat samples. Samples from S1 and S2 are marked in red, and samples from D1, D2 and D3 are marked in black. Samples at three different locations were respectively represented as squares, triangles and circles.

Table 4 shows the triples distance between the reference and the clustering trees. The best clustering tree was achieved by VLMC with 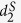 when *k* = 8 as shown in Fig. 5b, and the smallest triples distance is 76. Samples from surface water (S1, S2) and from deeper regions (D1, D2 and D3) are clearly separated. In contrast, the best result based on the FOMC background sequence model showed that surface samples were merged with deeper samples successively, as shown in Fig. 5c.

**Figure 6:**
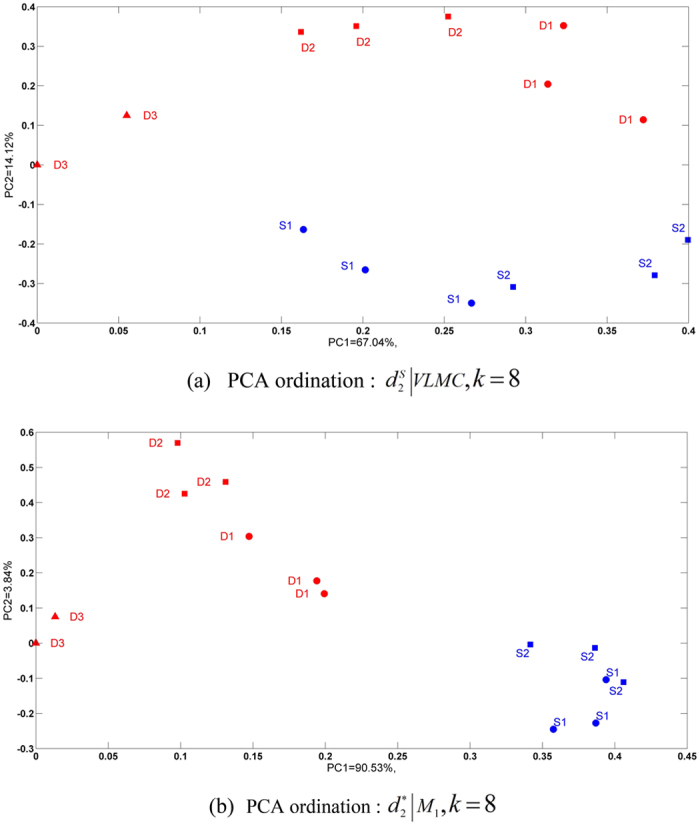
PCA ordinates of samples in Experiment 5. (**a**) Two-dimensional PCA plot based on FOMC. (**b**) Two-dimensional PCA plot based on VLMC.

The two-dimension Principal Component Analysis (PCA) plots based on the optimal results from FOMC and VLMC when *k* = 8 are shown in Fig. 6a,b, respectively. The PCA plot based on VLMC reflects the gradient information for collection depths and sites as the first and second principal component. We also plotted the PCA figures for *k* = 7 and 9, shown in Figure S3 in Supplementary Section 4. Although *k* = 7 and 9 are not the optimal value for VLMC, they still can separate the different depths and collecting sites. In comparison, the PCA ordinates based on FOMC did not show clear separations, and some points are shown as outliers.

## Discussion and Conclusions

In this study, we developed theoretical and computational approaches to model background sequences using VLMCs based on short reads from high throughput sequencing. We compared the performances of VLMC and FOMC with 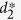 and 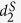, as well as *d*_2_ and three *L*_*p*_*-norm* measures, to model the background sequence with one simulated dataset, three real metatranscriptomic datasets, and one real RNA-seq dataset. VLMC outperformed FOMC in all experiments; and 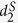 together with VLMC, as background sequence model, outperformed FOMC in all experiments. Experiments show that VLMC builds the model with adaptive and variable MC according to the metatranscriptomic data, exempting from manual selection of a fix MC order. Compared with FOMC, VLMC are more structural rich and easy to use. Based on the experimental results, we show that 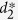 and 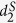 dissimilarity measures combined with VLMC background model can identify the underlying relationships among samples from different microbial communities. They can also reveal the gradient relationship among the samples. Therefore, such dissimilarity measures should be adopted in comparative transcriptomic and metatranscriptomic studies.

In this study, we only applied VLMC to RNA-Seq or metatranscriptomic datasets. We also attempted to apply VLMC to metagenomic datasets, but here, VLMC does not achieve obvious improvements compared with the results of FOMC. For instance, we applied VLMC to analyze a real mammalian gut metagenomic dataset^33^. It includes 21 samples from mammalian species of herbivores and 7 samples from species of carnivores. As shown in Figure S4 in Supplementary section 5, results indicate that VLMC is less effective than FOMC in distinguishing between the two mammalian sample types. This could be attributed to the inclusion of both expressed and non-expressed regions in the whole genomes, making them heterogeneous. One model cannot fit the data well resulting in a simple independent identically distributed yielding the most meaningful results in most cases. Since the transcriptome only includes expressed regions, they will most likely be homogeneous, and a Markov model may fit better. Thus, while VLMC can improve performance for metatranscriptomic datasets, it does not show obvious improved performance for metagenomic datasets.

Alignment-free method avoids the complications of alignment-based approach, and is able to process the microbial community with a large amount of dark matters. However, it does not provide detail insights of microbial communities and further biological interpretation. To answer such questions, alignment-based methods are still needed.

## Methods

### Processing flow chart

**Figure 7:**
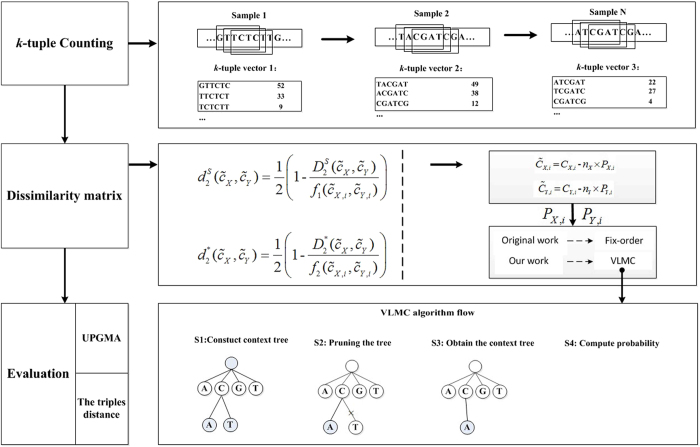
Flow chart of our approach: (1) The frequency vector of 1–10 tuples is generated from the sequencing data. (2) Markov transition probability of each tuple is calculated based on VLMC, and different dissimilarity measures are applied to *k*-tuple sequence signature. (3) Measured dissimilarities are evaluated. We used UPGMA for hierarchical clustering based on dissimilarity matrix and applied the triples distance to evaluate the consistency between the reference and the clustering trees.

The processing procedure consists of three main steps: (1) calculating *k-*tuple frequency; (2) calculating the probability of each tuple based on VLMC and applying various dissimilarity measures to *k-*tuple frequencies; and (3) evaluating different dissimilarity measures and models for background sequences. We used UPGMA^34^ for hierarchical clustering based on dissimilarity matrix and applied the triples distance^26^ to evaluate consistency between the reference tree and the clustering tree. We extended the VLMC algorithm to make it suitable for high-throughput sequencing data and then applied VLMC to model the underlying background genomes in 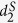 and 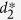
dissimilarity measures. Figure 7 shows the flow chart, and the details of these steps are given below.

### Calculating *k*-tuple frequency

Alignment-free methods use *k-*tuple frequencies as sequence signatures to represent each metatranscriptomic datum. In our study, *k*-tuple frequencies from *k* = 1 to a maximum *k* value are calculated with our developed pipeline, taking complementary strands into consideration. The maximum *k* value is *d* + 1, where *d* is the depth of the full prefix tree constructed in step (1). In our study, the depth of the prefix tree is 10. The *k*-tuple frequencies are used in constructing prefix tree, calculating the transition probabilities and compute dissimilarity measures.

### Dissimilarity measures based on *k*-tuple frequency

The dissimilarity between two samples is calculated based on the frequency vectors using various measures, including measures with background model normalization such as 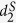 and 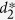 with VLMC/FOMC background sequence models, and measures without background model normalization such as *d*_2_, *Ma*, *Ch* and *Eu* in our study. The calculation of 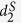 and 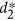 is described briefly as follows^21^:

Let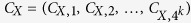 and 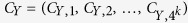 represent the *k-*tuple frequency vectors of sequencing data *X* and *Y*, Let 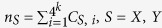 be the sum of the counts of all *k-*tuples. The 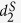 and 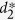 dissimilarity measures are defined in equations (1) and (2), where 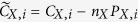 and 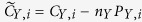. The ranges of 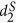 and 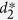 are between 0 and 1.


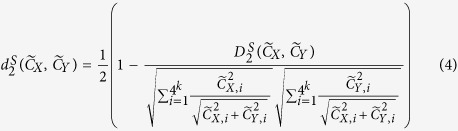



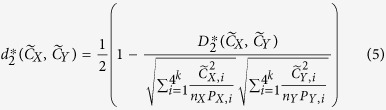


where *P*_*X,i*_ and *P*_*Y,i*_ are the probability of the *i*^*th*^ *k*-tuple based on *X* and *Y*, respectively. The probabilities are calculated based on a specific probabilistic model. For example, consider a 5-tuple “*GCTAC*”. Then P(*GCTAC*) can be calculated as:


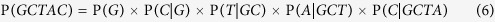


In previous studies^1^, FOMC was used to compute transition probability with fixed order *r*. For example, when *r* = 2,


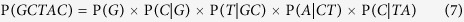


In application, the order of MC needs to be set manually. But for most microbial communities, there is no prior knowledge available for MC order. Furthermore, it is hard to model probabilities of different tuples using a single fixed order MC. Variable Length Markov Chains^24^ (VLMC) model the background genomes selecting the MC order adaptively in a data-driven way. For example, the probability (3) might be represented as formula (8) after determining the order in VLMC:


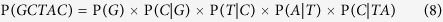


Thus VLMC is more structurally rich and the number of variables is flexible. VLMC was originally designed to model long sequences^24^,^35^ and was represented as a context tree structure^25^. In our study, VLMC was extended to model the background genomes based on short reads from high throughput sequencing.

### VLMC for modeling background genomes with high-throughput sequencing data

**Figure 8:**
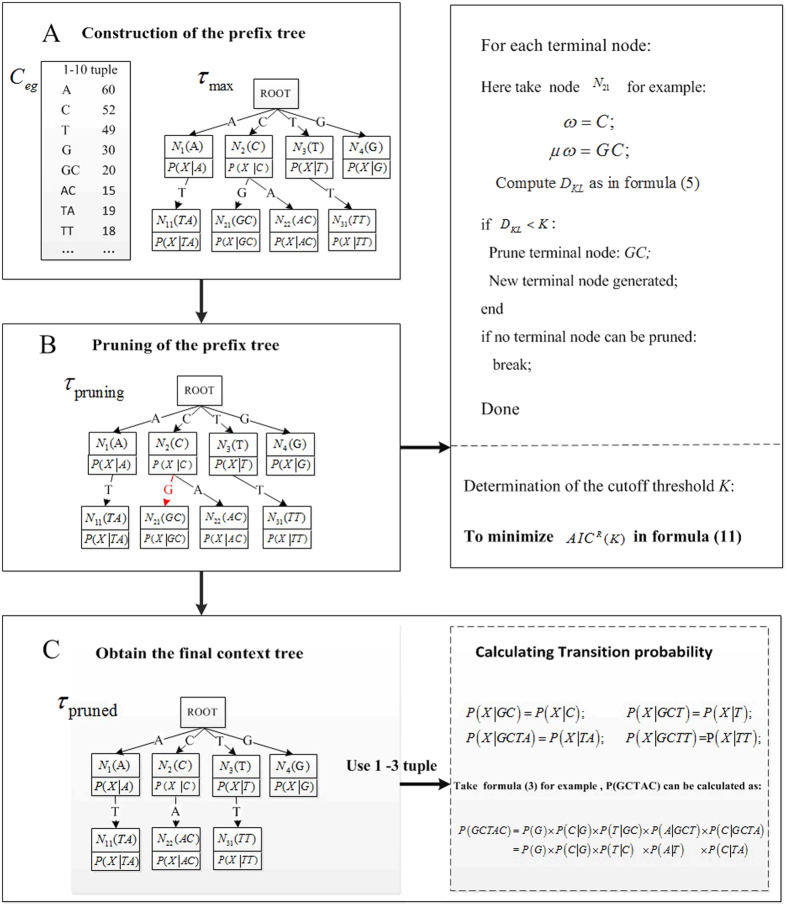
Flow chart showing the construction of VLMC based on high-throughput sequencing data. (**A**) Construction of the prefix tree. (**B**) Pruning of the prefix tree. (**C**) Calculation of probability based on the pruned (or context) tree.

The VLMC for high-throughput sequencing data is implemented with the following three steps: (1) A full prefix tree is built based on 1, 2, …, 10-tuple frequency vectors, but the tree usually overfits the data. (2) The tree is subsequently pruned to remove redundant branches based on Kullback-Leibler divergence^36^, and the pruned tree is also called a context tree^25^. (3) Transition probabilities are calculated with respect to the MC orders from the context tree, and the probabilities of *k*-tuples are then computed accordingly. A specific example is given in Fig. 8. The three steps were inspired by the original VLMC method on a single genomic sequence proposed by Bühlmann P. and Wyner, A. J. in 1999^24^.

### Step 1: Generating a prefix tree *τ*_*max*_ based on tuple frequency

We first generate a tree *τ*_*max*_ to store tuples in the frequency vector. The tree *τ*_*max*_ is actually a prefix tree growing downwards, where each node in the tree represents a tuple. The *l*^th^ level nodes represent tuples of length *l*. In our study, the maximum depth of the tree *τ*_*max*_ is up to 10. The following logic determines the relationships connecting nodes. If a node represents the *l*-tuple *ω* ∈{*A,C,G,T*}*l*, *l* = 1, 2, …, 9, then its offspring represents the (*l* + 1)-tuple word *μω (μ* is a character in front of *ω*, *μ* ∈{*A,C,G,T*}. For a node representing *ω*, the transition probability is calculated as P_*X*_(*X*|*ω*) = *C*_*X,*__*ωX*_/*C*_*X,*__*ω*_ and saved at the node. In practice, the construction of *τ*_*max*_ based on 1, 2, …, *k*-tuple frequency vectors is fast.

In Fig. 8A, *τ*_*max*_ is generated based on frequency vector *C*_*gg*_. Node *N*_2_(*C*) represents tuple *C*, and its offspring *N*_21_(*GC*) represents tuple *GC*. Additionally, each node is associated with the transition probability from corresponding tuple to *X (X*∈{*A*,*C,G,T*}). Node *N*_2_(*C*) is associated with P(*X*|*C*), and node *N*_21_(*GC*) is associated with P(*X*|*GC*).

### Step 2: Pruning the tree *τ*_*max*_

The next step involves pruning the tree *τ*_*max*_ to remove redundant branches. If the probability P(*X*|*μω*) for a terminal node *μω* is the same as its parent node’s transition probability P(*X*|*ω*), meaning that the transition probability of *μω* can be replaced by that of *ω*, then the terminal node *μω* can be pruned from the branch. In our study, Kullback-Leibler divergence is a measure of the distance between two probability distributions P(*X*|*μω*) and P(*X*|*ω*). Accordingly, Kullback-Leibler divergence^36^ is applied to compare P(*X*|*μω*) and P(*X*|*ω*), which is denoted as *D*_*KL*_(*P(X*|*μω*)||P(*X*|*ω*)). A value of *D*_*KL*_(*P(X*|*μω*)||P(*X*|*ω*)) less than a threshold value *K* indicates that no information is lost when P(*X*|*ω*) is used to approximate P(*X*|*μω*), thereby allowing *μω* to be pruned. *D*_*KL*_(*P(X*|*μω*)||P(*X*|*ω*)) is given by formula (9), and *N*(*) is the frequency.


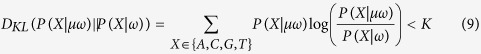



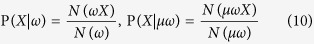


Taking Fig. 8B as an example, the Kullback-Leibler divergence between *N*_21_(*GC*) and *N*_2_(*C*) is calculated to determine whether node*N*_21_(*GC*) should be pruned:


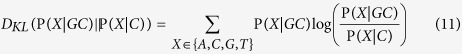


Suppose that threshold *K* is set to 5, then node *N*_21_(*GC*) should be pruned if


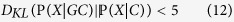


The pruning is implemented for each terminal node until no branches can be pruned. *K* is the threshold that determines the degree of pruning. A larger *K* means greater conditional latitude in branch pruning, in turn producing a smaller tree.

Similar to the study of Mächler and Bühlmann^35^, the determination of *K* is implemented through the optimization of Akaike Information Criterion (AIC)^37^ designed for high-throughput sequencing data. AIC measures the relative quality of statistical models for a given set of data. AIC is originally defined as


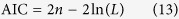


where *L* is the maximum value of the likelihood function for a statistical model, indicating the goodness of fit of the model, and *n* is the number of parameters in the model, indicating the complexity of the model.

Here we develop the AIC calculation algorithm for high-throughput sequencing data. Given high-throughput sequencing data with *M* reads of length *β*,


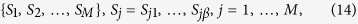


where *S*_*j*_ is the *j*^th^ read, *S*_*ji*_ is the *i*^th^ nucleotide of *j*^th^ read, and *S*_*ji*_ ∈ {*A*,*C*,*G*,*T*}. Then, AIC with pruning threshold *K* is defined as:


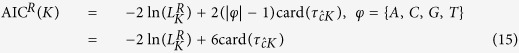


where card (*τ*_*ĉK*_) denotes the number of nodes in the context tree *τ*_*ĉK*_, and 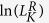
is the log-pseudo-likelihood under a fitted VLMC model with threshold *K*. The superscript *R* denotes the short read data. The log-pseudo-likelihood of the sequencing data is


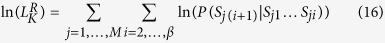


where P(*S*_*j(i*+ 1)_|*S*_*j*1_…*S*_*ji*_) is the estimated transition probability from the high-throughput sequencing data. The optimal *K* is determined by minimizing the formula AIC^*R*^(*K*).

The two steps of tree building and pruning for high throughput sequencing data is extended from the original algorithm for long sequences from Bühlmann *et al.*^24^. The pruning step starts from the terminal nodes and the procedure is repeated until no more pruning is possible. The algorithm is greedy, so it is possible that the final pruned context tree is not the global optimal one. The R-package for long sequences^35^ developed in 2012 follows the same greedy algorithm.

### Step 3: Calculating probabilities of tuples based on the context tree

The corresponding probabilities of tuples are calculated based on the context tree. The number of independent parameters is Num(nodes) × 3, where the Num(nodes) is the total number of nodes in the context tree except the root-node. Taking the context tree in Fig. 8C as an example, Node *N*_21_(G*C*) was pruned away; therefore, in tuple *GCX, G* has no effect on the transition probability from *GC* to state *X*. Thus, P(*X*|*GC*) can be replaced by P(*X*|*C*) and stored in node *N*_2_(*C*) of the context tree in Fig. 8C.


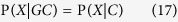


The tuples in the node of context tree can be of variable length, allowing the VLMC model to estimate the transition probability. The corresponding probabilities of tuples used in 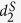 and 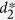 are then computed based on the transition probabilities. For example in Fig. 8C, the probability of 5-tuple word “*GCTAC*”,


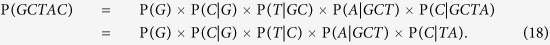


In the real data from marine metatranscriptome, there are ~10^3^ nodes for the pruned context tree with 8 levels and ~10^2^ nodes for the tree with 7 levels, which means that the number of parameters reduced from 4^8^ × 3∼2 × 10^5^ to ∼10^3–4^ for *r* = 8; and from 4^7^ × 3∼5 × 10^4^ to ∼10^2–3^ for *r* = 7, at least 10-fold decrease in the number of parameters.

### Using a heuristic approach to search for optimal *K*

**Figure 9:**
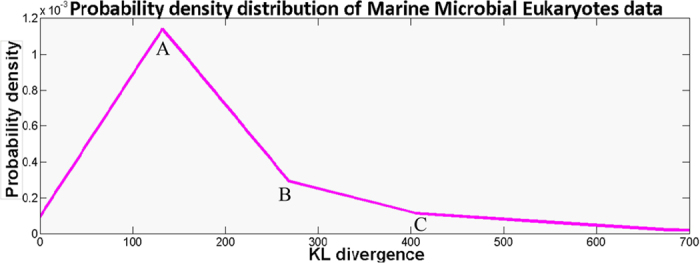
Probability density distributions of 22 Marine Microbial Eukaryotes RNA-Seq data.

The value of *K* is determined by minimizing AIC^*R*^(*K*). However, no simple analytical formula exists between *K* and AIC^*R*^(*K*), making it a challenge to find the optimal *K* for all sequencing data. To solve this problem, we developed the following heuristic approach to determine the value of *K*. In our study, one branch is pruned when its Kullback-Leibler divergence is less than the threshold *K*. Therefore, *K* is meaningful only when it is within the value range of Kullback-Leibler divergence. In Experiment on 22 Marine Microbial Eukaryotes, the probability density distribution of the Kullback-Leibler divergence is shown as Fig. 9. The values of Kullback-Leibler divergence in most tuples are between 100 and 500. Optimal results are generally obtained with *K* setting around the peak points and the right two inflexions (point A, B and C in Fig. 9). Hence, we only implement local search around these three points for the optimal *K* that minimizes loss functionAIC^*R*^(*K*).

**Sample clustering with UPGMA^34^ (Unweighted Pair Group Method with Arithmetic Mean)** is a hierarchical clustering method initially designed for classification problems. UPGMA is now widely used for hierarchical clustering in bioinformatics based on dissimilarity matrices. The nearest two clusters are combined into a higher-level cluster. The distance between two clusters *A* and *B* is defined as the average of all distances between pairs of samples *x* in *A* and *y* in *B*. The calculation is presented in equation (14), where *d (x*,*y*) refers to the dissimilarity between sample *x* and sample *y*. This is repeated for each step. UPGMA is implemented with the function ‘*upgma*’ from the ‘*phangorn*’ toolbox of *R*.


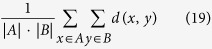


**The selection of proper evaluation metrics:** Based on the dissimilarity matrix from different background models, the hierarchical clustering trees are produced. The consistency between the reference and the clustering trees offers the metrics to evaluate the performance of the various background models. There are several metrics to measure the difference of topological structures between two trees.

**Parsimony score^38^,^39^** is the most common one to compare the topological structures of two trees. The parsimony score for a tree is the sum of the smallest number of substitutions needed comparing with the reference tree, which was implemented with toolbox *Mothur* in our study. When one tree is binary and one tree is not binary, the parsimony score is not suitable for comparison of the trees.

**Symmetric difference^40^** was originally defined to compare two node sets. It has been used as a criterion to evaluate the consistency between two trees^38^. Two trees *A* and *B* have the same leaves, and their node sets are 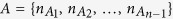 and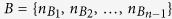. The symmetric difference between *A* and *B* is defined as


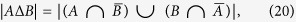


i.e., the set of nodes present in one tree, but not in the other tree, where |*| is the number of elements, and 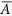 and 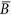 are the complements of set *A* and set *B*, respectively. Compared with the parsimony score, symmetric difference does not use branch length information, only tree topologies. Moreover, symmetric difference has taken the order of hierarchical clustering into consideration, making the comparison more sensitive. Symmetric difference is calculated with *Treedist* from *Phylip*.

**The triples distance^26^**, another tree comparison metric to measure the distance between binary^26^ or non-binary trees^41^, is also used. In our study, some reference trees are rooted non-binary trees. The measures are based on the topologies of the input trees induce on triplets; that is, on three-element subsets of the set of species. Triplet based distances provide a robust and fine-grained measure of the similarities between trees^41^, which was developed as toolbox *TreeCmp*^42^.

The above three metrics have different characteristics and application scopes of their own. In Supplementary Section 7, we constructed example trees and measure their distances with the three metrics. Table S6 shows the three metrics for experiment 5, and the three metrics reflect general consistent tendency of tree distance. These two experiments show that the triples distance is most suitable and has high accuracy to evaluate the consistence of topologies of two trees.

**Principal component analysis (PCA)^43^** is an important tool to analyze a multivariate data table in which observations are described by several inter-correlated quantitative dependent variables. Its goal is to extract the important information from the table and to represent the information as a set of new orthogonal variables called principal components. In *R* ‘*ape*’ toolbox, the functions *princomp* and *prcomp* can be used for principal component analysis.

## Additional Information

**Accession codes**: https://d2vlmc.codeplex.com.

**How to cite this article**: Liao, W. *et al.* Alignment-free Transcriptomic and Metatranscriptomic Comparison Using Sequencing Signatures with Variable Length Markov Chains. *Sci. Rep.* **6**, 37243; doi: 10.1038/srep37243 (2016).

**Publisher's note:** Springer Nature remains neutral with regard to jurisdictional claims in published maps and institutional affiliations.

## Supplementary Material

Supplementary Information

## Figures and Tables

**Table 1:** The triples distance between the reference and the clustering trees using various background models with *k* = 2–9 for the simulation dataset of Experiment 1.

	*k*	2	3	4	5	6	7	8	9
VLMC	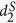	91845	90796	91209	90583	89748	74759	54841	***42973***
	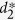	92398	91368	91967	91557	90858	91088	92198	91266
FOMC	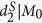	91737	91317	90938	89990	89176	73161	64770	58339
	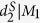	91845	90825	89631	89432	86202	74615	66449	63143
	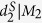	NA	91215	90772	89205	88783	77420	61034	46369
	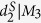	NA	NA	91244	90672	89128	67519	62031	***43047***
	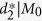	91799	91887	90788	90149	89170	83466	64932	56770
	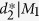	92398	90617	88314	90011	86425	73498	63022	59554
	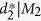	NA	90684	90589	87693	86522	75092	58091	50969
	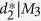	NA	NA	91002	89840	89773	62601	55456	50293
*L*_*p*_ *-Norm* and *d*_2_ measures	*d*_2_	90569	89957	88967	88062	84338	72034	65577	63569
	*Ch*	89553	89271	90366	89440	91424	90956	90456	90913
	*Eu*	90800	90930	89777	88271	86178	74766	67672	66107
	*Ma*	90338	90835	88814	89461	85528	71241	50671	47623

“*M*_*i*_” indicates that the expected counts are calculated based on an *i*-th order Markov model for the background sequences. The p-values are estimated by comparing the observed triples distance to the triples distance in 3000 randomly-joined trees: for triples distance < 8000: p < 0.001.

**Table 2:** The triples distance between the reference and the clustering trees using various background models with *k* = 2–9 for the 18 RNA-Seq data in Experiment 2.

	*k*	2	3	4	5	6	7	8	9
VLMC	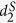	354	375	415	193	***177***	241	227	219
	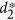	359	421	236	187	240	293	291	279
FOMC	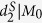	336	327	407	407	403	428	428	428
	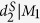	550	582	504	518	518	518	456	487
	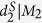	NA	561	515	533	518	506	489	476
	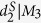	NA	NA	534	581	577	509	500	440
	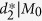	***318***	326	410	415	456	526	521	548
	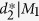	537	557	565	568	592	592	585	541
	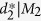	NA	561	533	535	566	570	535	500
	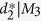	NA	NA	554	573	564	545	526	506
*L*_*p*_ *-Norm* and d_2_ measures	*d*_2_	466	475	470	480	480	504	492	553
	*Ch*	491	490	499	523	546	579	556	567
	*Eu*	470	474	474	479	494	564	564	530
	*Ma*	495	473	473	480	472	474	478	480

“*M*_*i*_” indicates that the expected counts are calculated based on an i-th order Markov model for the background sequences. The p-values are estimated by comparing observed the triples distance to the triples distance in 3000 randomly-joined trees: for triples distance in 300~400: p < 0.006; for the triples distance < 300: p < 0.001.

**Table 3:** The triples distance between the reference and the clustering trees using various background models with *k* = 2–9 to identify the gradient relationships of metagenomic and metatranscriptomic samples at different ocean depths in Experiment 4.

	*k*	2	3	4	5	6	7	8	9
VLMC	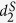	131	8	16	16	***0***	4	30	131
	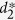	123	24	16	***0***	***0***	12	156	123
FOMC	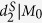	32	24	32	16	16	16	16	32
	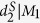	131	40	32	16	16	16	***8***	131
	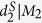	NA	40	*16*	***8***	***8***	***8***	***8***	40
	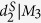	NA	NA	34	***8***	***8***	***8***	***8***	34
	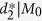	40	32	32	32	24	24	24	40
	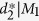	123	16	16	16	***8***	***8***	***8***	123
	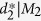	NA	24	**8**	***8***	***8***	***8***	***8***	24
	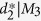	NA	NA	16	***8***	***8***	***8***	***8***	16
*L*_*p*_ *–Norm and d*_2_ measures	*d*_2_	112	32	32	32	32	32	32	112
	*Ch*	32	128	128	128	136	136	128	32
	*Eu*	112	32	32	32	32	32	40	112
	*Ma*	112	112	16	16	16	16	16	112

“*M*_*i*_” indicates that the expected counts are calculated based on an i-th order Markov model for the background sequences. The p-values are estimated by comparing observed the triples distance to the triples distance in 3000 randomly-joined trees: for all triples distance in table, p < 0.001.

**Table 4:** The triples distance between the reference and the clustering trees using various background models with *k* = 2–9 to identify the gradient relationships of metatranscriptomic samples of microbial mats in Experiment 5.

	*k*	2	3	4	5	6	7	8	9
VLMC	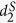	251	251	283	274	245	195	***76***	***76***
	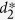	251	274	273	263	228	210	***76***	262
FOMC	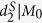	218	189	189	202	202	178	201	189
	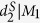	251	248	159	202	201	178	201	189
	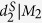	NA	276	154	197	201	178	201	189
	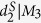	NA	NA	272	201	118	164	170	189
	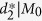	251	222	221	159	202	178	178	***148***
	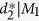	251	218	159	202	195	178	***148***	***148***
	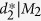	NA	274	154	154	195	178	***148***	***148***
	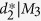	NA	NA	268	201	195	***148***	***148***	***148***
*L*_*p*_ *-Norm* and d_2_ measures	*d*_2_	264	218	213	159	198	178	178	***148***
	*Ch*	264	247	256	260	241	207	232	238
	*Eu*	264	218	213	159	198	165	162	162
	*Ma*	264	218	213	159	198	206	206	202

*“M*_*i*_” indicates that the expected counts are calculated based on an i-th order Markov model for the background sequences. The p-values are estimated by comparing the observed triples distance to the triples distance in 3000 randomly-joined trees: for the triples distance < 155:p < 0.001.

## References

[b1] Wang Y., Liu L., Chen L., Chen T. & Sun F. Comparison of metatranscriptomic samples based on k-tuple frequencies. PloS One 9, e84348 (2014). 24392128 10.1371/journal.pone.0084348 PMC3879298

[b2] Smith T. F. & Waterman M. S. Identification of common molecular subsequences. Journal of Molecular Biology 147, 195–197 (1981). 7265238 10.1016/0022-2836(81)90087-5

[b3] Altschul S. F., Gish W., Miller W., Myers E. W. & Lipman D. J. Basic local alignment search tool. Journal of Molecular Biology 215, 403–410 (1990). 2231712 10.1016/S0022-2836(05)80360-2

[b4] Wood D. E. & Salzberg S. L. Kraken: ultrafast metagenomic sequence classification using exact alignments. Genome Biology 15 (2014). 10.1186/gb-2014-15-3-r46 PMC4053813 24580807

[b5] Ounit R., Wanamaker S., Close T. J. & Lonardi S. CLARK: fast and accurate classification of metagenomic and genomic sequences using discriminative k-mers. BMC Genomics 16 (2015). 10.1186/s12864-015-1419-2 PMC4428112 25879410

[b6] Menzel P., Ng K. L. & Krogh A. Fast and sensitive taxonomic classification for metagenomics with Kaiju. Nature Communications 7, 11257 (2016). 10.1038/ncomms11257 PMC4833860 27071849

[b7] Segata N. *et al.* Metagenomic microbial community profiling using unique clade-specific marker genes. Nature Methods 9, 811–814 (2012). 22688413 10.1038/nmeth.2066 PMC3443552

[b8] Shi Y., Tyson G. W. & DeLong E. F. Metatranscriptomics reveals unique microbial small RNAs in the ocean’s water column. Nature 459, 266–226 (2009). 19444216 10.1038/nature08055

[b9] Leimena M. M., Ramiro-Garcia J. & Davids M. A comprehensive metatranscriptome analysis pipeline and its validation using human small intestine microbiota datasets. BMC Genomics 14, 530 (2013). 23915218 10.1186/1471-2164-14-530 PMC3750648

[b10] Adria M., David M. S. & Colleen A. D. Comparative metatranscriptomics identifies molecular bases for the physiological responses of phytoplankton to varying iron availability[J]. Proceedings of the National Academy of Sciences 109, 317–325 (2012). 10.1073/pnas.1118408109 PMC3277525 22308424

[b11] Martinez X. *et al.* MetaTrans: an open-source pipeline for metatranscriptomics. Scientific Reports 6, 26447 (2016). 27211518 10.1038/srep26447 PMC4876386

[b12] Frazee A. C., Jaffe A. E., Langmead B. & Leek J. T. Polyester: simulating RNA-seq datasets with differential transcript expression. Bioinformatics 31, 2778–2784 (2015). 25926345 10.1093/bioinformatics/btv272 PMC4635655

[b13] Lippert R. A., Huang H. & Waterman M. S. Distributional regimes for the number of k-word matches between two random sequences. Proceedings of the National Academy of Sciences 99, 13980–13989 (2002). 10.1073/pnas.202468099 PMC137823 12374863

[b14] Karlin S., Mrazek J. & Campbell A. M. Compositional biases of bacterial genomes and evolutionary implications. Journal of Bacteriology 179, 3899–3913 (1997). 9190805 10.1128/jb.179.12.3899-3913.1997 PMC179198

[b15] Reinert G., Chew D., Sun F. & Waterman M. S. Alignment-free sequence comparison (I): statistics and power. Journal of Computational Biology 16, 1615–1634 (2009). 20001252 10.1089/cmb.2009.0198 PMC2818754

[b16] Kantorovitz M. R., Robinson G. E. & Sinha S. A statistical method for alignment-free comparison of regulatory sequences. Bioinformatics 23, i249–i255 (2007). 17646303 10.1093/bioinformatics/btm211

[b17] Wan L., Reinert G., Sun F. & Waterman M. S. Alignment-free sequence comparison (II): theoretical power of comparison statistics. Journal of Computational Biology 17, 1467–1490 (2010). 20973742 10.1089/cmb.2010.0056 PMC3123933

[b18] Dai Q. & Wang T. Comparison study on k-word statistical measures for protein: From sequence to ‘sequence space’. BMC Bioinformatics 9, 394 (2008). 18811946 10.1186/1471-2105-9-394 PMC2571980

[b19] Dai Q., Yang Y. & Wang T. Markov model plus k-word distributions: a synergy that produces novel statistical measures for sequence comparison. Bioinformatics 24, 2296–2302 (2008). 18710871 10.1093/bioinformatics/btn436

[b20] Qi J., Wang B. & Hao B. L. Whole proteome prokaryote phylogeny without sequence alignment: a K-string composition approach. Journal of Molecular Evolution 58, 1–11 (2004). 14743310 10.1007/s00239-003-2493-7

[b21] Song K. *et al.* Alignment-free sequence comparison based on next-generation sequencing reads. Journal of Computational Biology 20, 64–79 (2013). 23383994 10.1089/cmb.2012.0228 PMC3581251

[b22] Jiang B. *et al.* Comparison of metagenomic samples using sequence signatures. BMC Genomics 13, 730 (2012). 23268604 10.1186/1471-2164-13-730 PMC3549735

[b23] Ren J., Song K., Deng M. & Reinert G. Inference of Markovian properties of molecular sequences from NGS data and applications to comparative genomics. Bioinformatics 32, 993–1000 (2016). 26130573 10.1093/bioinformatics/btv395 PMC6169497

[b24] Bühlmann P. & Wyner A. J. Variable length Markov chains. The Annals of Statistics 27, 480–513 (1999).

[b25] Rissanen J. A universal data compression system. IEEE Transactions On Information Theory 29, 656–664 (1983).

[b26] Critchlow D. E., Pearl D. K. & Qian C. The triples distance for rooted bifurcating phylogenetic trees. Systematic Biology 45, 323–334 (1996).

[b27] Duanmu D. *et al.* Marine algae and land plants share conserved phytochrome signaling systems. Proceedings of the National Academy of Sciences 111, 15827–15832 (2014). 10.1073/pnas.1416751111 PMC4226090 25267653

[b28] Huelsenbeck J. P. & Ronquist F. MRBAYES: Bayesian inference of phylogenetic trees. Bioinformatics 17, 754–755 (2001). 11524383 10.1093/bioinformatics/17.8.754

[b29] Qin J. *et al.* A human gut microbial gene catalogue established by metagenomic sequencing. Nature 464, 59–65 (2009). 10.1038/nature08821 PMC3779803 20203603

[b30] Keeling P. J. *et al.* The Marine Microbial Eukaryote Transcriptome Sequencing Project (MMETSP): illuminating the functional diversity of eukaryotic life in the oceans through transcriptome sequencing. PLoS Biol 12(6), e1001889 (2014). 24959919 10.1371/journal.pbio.1001889 PMC4068987

[b31] Karl D., Bidigare R. & Letelier R. Long-term changes in plankton community structure and productivity in the North Pacific Subtropical Gyre: the domain shift hypothesis. Deep Sea Research Part II: Topical Studies in Oceanography 48, 1449–1470 (2001).

[b32] Quaiser A. *et al.* Unraveling the stratification of an iron-oxidizing microbial mat by metatranscriptomics. PLoS One 9(7) e102561 (2014). 25033299 10.1371/journal.pone.0102561 PMC4102501

[b33] Muegge B. D., Kuczynski J. & Knights D. Diet drives convergence in gut microbiome functions across mammalian phylogeny and within humans. Science 332, 970–974 (2011). 21596990 10.1126/science.1198719 PMC3303602

[b34] Murtagh F. Complexities of hierarchic clustering algorithms: State of the art. Computational Statistics Quarterly 1, 101–113 (1984).

[b35] Mächler M. & Bühlmann P. Variable length Markov chains: methodology, computing, and software. Journal of Computational and Graphical Statistics 13(2), 435–455 (2012).

[b36] Kullback S. & Leibler R. A. On Information and Sufficiency. Annals of Mathematical Statistics 22, 79–86 (1951).

[b37] Akaike H. Factor analysis and AIC. Psychometrika 52, 317–332 (1987).

[b38] Robinson D. & Foulds L. R. Comparison of phylogenetic trees. Mathematical Biosciences 53, 131–147 (1981).

[b39] Schloss P. D. & Handelsman J. Introducing TreeClimber, a test to compare microbial community structures. Applied and Environmental Microbiology 72, 2379–2384 (2006). 16597933 10.1128/AEM.72.4.2379-2384.2006 PMC1449046

[b40] Penny D. & Hendy M. The use of tree comparison metrics. Systematic Zoology 34, 75–82 (1985).

[b41] Bansal M. S., Dong J. & Fernández-Baca D. Comparing and aggregating partially resolved trees. Theoretical Computer Science 412, 6634–6652 (2011).

[b42] Bogdanowicz D., Giaro K. & Wróbel B. TreeCmp: Comparison of Trees in Polynomial Time. Evolutionary Bioinformatics Online 8, 475–487 (2012).

[b43] Wold S., Esbensen K. & Geladi P. Principal component analysis. Chemometrics and Intelligent Laboratory Systems 2, 37–52 (1987).

