# Supplementary material for: Alignment-free Transcriptomic and Metatranscriptomic Comparison Using Sequencing Signatures with Variable Length Markov Chains
Source: Sci Rep. 2016 Nov 23;6:37243. doi: 10.1038/srep37243 (PMC5120338; doi:10.1038/srep37243)
Supplement: Supplementary Information [file srep37243-s1.pdf]

Supplementary Information

Title of manuscript:

Alignment-free Transcriptomic and Metatranscriptomic Comparison Using Sequencing Signatures with Variable Length Markov Chains

Authors:

Weinan Liao[1†], Jie Ren[2†], Kun Wang[1], Shun Wang[1], Feng Zeng[1], Ying Wang[1*], Fengzhu Sun[2*]

Supplementary information includes:

Supplementary Tables S1-S6

Supplementary Figures S1-S6

Supplementary Reference

1. Comparison based on RNA-Seq data of Marine Microbial Eukaryotes in Experiment 2

1.1 Comparison based on 18 RNA-Seq data of Marine Microbial Eukaryotes in Experiment 2

Table S1. Reference information for 18 RNA-seq[1] data in Experiment 2

SRA	Strain	18S rRNA	Organisms	Kingdom	Phylum	Class
SRR1300224	*CCMP1194*	*AF203400*	*Prasinococcus sp.*	*Eukaryota*	*Chlorophyta*	*Prasinophyceae*
SRR1296862	*CCMP1413*	*FN562437*	*Prasinoderma coloniale*	*Eukaryota*	*Chlorophyta*	*Prasinophyceae*
SRR1300428	*CCMP720*	*KF615764*	*Polyblepharides amylifera*	*Eukaryota*	*Chlorophyta*	*Prasinophyceae*
SRR1296737	*CCMP725*	*KF615765*	*Pyramimonas parkeae*	*Eukaryota*	*Chlorophyta*	*Prasinophyceae*
SRR1296860	*CCMP3274*	*KF615766*	*Dolichomastix tenuilepAis*	*Eukaryota*	*Chlorophyta*	*Mamiellophyceae*
SRR1296861	*CCMP3273*	*KF615767*	*Crustomastix stigmatica*	*Eukaryota*	*Chlorophyta*	*Mamiellophyceae*
SRR3384777	*CCMP1545*	*AY954994*	*Micromonas pusilla*	*Eukaryota*	*Chlorophyta*	*Mamiellophyceae*
SRR3384320	*RCC299*	*HM191693*	*Micromonas sp.*	*Eukaryota*	*Chlorophyta*	*Mamiellophyceae*
SRR1300441	*CCMP2099*	*AY954999*	*Micromonas sp.*	*Eukaryota*	*Chlorophyta*	*Mamiellophyceae*
SRR1300455	*CCAC1681*	*FN562452*	*Micromonas pusilla*	*Eukaryota*	*Chlorophyta*	*Mamiellophyceae*
SRR1300295	*CCMP1646*	*AY954996*	*Micromonas sp.*	*Eukaryota*	*Chlorophyta*	*Mamiellophyceae*
SRR1300453	*CCMP1898*	*JX625115*	*Bathycoccus prasinos*	*Eukaryota*	*Chlorophyta*	*Mamiellophyceae*
SRR1294419	*CCMP717*	*KF615768*	*Nephroselmis pyriformis*	*Eukaryota*	*Chlorophyta*	*Nephroselmidophyceae*
SRR1300322	*CCMP1897*	*KF615769*	*Picocystis salinarum*	*Eukaryota*	*Chlorophyta*	*Picocystis salinarum*
SRR1300422	*CCMP1998*	*KF615770*	*Pycnococcus sp.*	*Eukaryota*	*Chlorophyta*	*Pycnococcaceae*
SRR1296698	*CCMP880*	*JN376804*	*Tetraselmis astigmatica*	*Eukaryota*	*Chlorophyta*	*Chlorodendraceae*
SRR1294459	*SAG11-48b*	*KF615772*	*Chlamydomonas chlamydogama*	*Eukaryota*	*Chlorophyta*	*Chlorophyceae*
SRR1294458	*SAG-1149*	*KF615771*	*Chlamydomonas leiostraca*	*Eukaryota*	*Chlorophyta*	*Chlorophyceae*

1.2 Comparison based on 22 RNA-Seq data of Marine Microbial Eukaryotes in Experiment 2

RNA-Seq data of 22 marine eukaryotes were downloaded from "The Marine Microbial Eukaryote Transcriptome Sequencing Project". Sample information of these 22 eukaryotes is listed in Table S2. These eukaryotes have a clear phylogenetic evolution hierarchy. The phylogenetic tree of these 22 RNA-Seq data is built with MrBayes, which is a program for Bayesian inference and model choice across a wide range of phylogenetic and evolutionary models. The phylogenetic tree is built according to the following steps: First 18s rRNA sequences of 22 species are downloaded from Silva database https://www.arb-silva.de/. Multiple alignment of the 18s rRNA sequences is done by Sliva as well, and then MrBayes is used to build phylogenetic trees based on multiple alignment using the default settings. Finally

the tree is plotted using R package "ape" in the unrooted type. The phylogenetic tree of these 22 RNA-Seq data is represented in Figure S1A.

Table S2. Reference information of 22 Marine Microbial Eukaryotes in Experiment 2

SRA	Sample Name	Abbreviation	Organism11s	Kingdom	Phylum	Class
SRS618835	MMETSP0397	*Ba-Fr-Cy*	*Cyclophora tenuis*	*Eukaryota*	*Bacillariophyta*	*Fragilariophyceae*
SRS626644	MMETSP0693	*Ba-Fr-Th*	*Thalassionema nitzschioides*	*Eukaryota*	*Bacillariophyta*	*Fragilariophyceae*
SRS619074	MMETSP0009_2	*Ba-Fr-Gr*	*Grammatophora oceanica*	*Eukaryota*	*Bacillariophyta*	*Fragilariophyceae*
SRS619075	MMETSP0902	*Ba-Co-Th*	*Thalassiosira antarctica*	*Eukaryota*	*Bacillariophyta*	*Coscinodiscophyceae*
SRS616853	MMETSP0010_2	*Ba-Co-Co*	*Corethron hystrix*	*Eukaryota*	*Bacillariophyta*	*Coscinodiscophyceae*
SRS616855	MMETSP0013_2	*Ba-Co-Sk*	*Skeletonema costatum*	*Eukaryota*	*Bacillariophyta*	*Coscinodiscophyceae*
SRS621600	MMETSP1437	*Ba-Me-Eu*	*Eucampia antarctica*	*Eukaryota*	*Bacillariophyta*	*Mediophyceae*
SRS616865	MMETSP1002	*Ba-Me-Di*	*Ditylum brightwellii*	*Eukaryota*	*Bacillariophyta*	*Mediophyceae*
SRS616857	MMETSP0015_2	*Ba-Me-Od*	*Odontella aurita*	*Eukaryota*	*Bacillariophyta*	*Mediophyceae*
SRS621548	MMETSP1404	*Ch-Ma-Mi*	*Micromonas pusilla*	*Eukaryota*	*Chlorophyta*	*Mamiellophyceae*
SRS621593	MMETSP1460	*Ch-Ma-Ba*	*Bathycoccus prasinos*	*Eukaryota*	*Chlorophyta*	*Mamiellophyceae*
SRS621618	MMETSP1468	*Ch-Ma-Ma*	*Mantoniella*	*Eukaryota*	*Chlorophyta*	*Mamiellophyceae*
SRS621597	MMETSP1459	*Ch-Py-Py1*	*Pycnococcus provasolii*	*Eukaryota*	*Chlorophyta*	*Pycnococcaceae*
SRS621453	MMETSP1316	*Ch-Py-Py2*	*Pycnococcus provasolii*	*Eukaryota*	*Chlorophyta*	*Pycnococcaceae*
SRP042159	MMETSP1085	*Ch-Py-Py3*	*Pycnococcus*	*Eukaryota*	*Chlorophyta*	*Pycnococcaceae*
SRS621414	MMETSP1161	*Ch-Tr-Pi1*	*Picochlorum oklahomense*	*Eukaryota*	*Chlorophyta*	*Trebouxiophyceae*
SRS621479	MMETSP1330	*Ch-Tr-Pi2*	*Picochlorum*	*Eukaryota*	*Chlorophyta*	*Trebouxiophyceae*
SRS616859	MMETSP0038_2	*Cr-Cr-Crp*	*Cryptomonas paramecium*	*Eukaryota*	*Cryptophyta*	*Cryptomonadaceae*
SRS621448	MMETSP1050	*Cr-Cr-Crc*	*Cryptomonas curvata*	*Eukaryota*	*Cryptophyta*	*Cryptomonadaceae*
SRS621446	MMETSP1049	*Cr-Ge-Pr*	*Proteomonas sulcata*	*Eukaryota*	*Cryptophyta*	*Geminigeraceae*
SRS621409	MMETSP1102	*Cr-Ge-Ge1*	*Geminigera*	*Eukaryota*	*Cryptophyta*	*Geminigeraceae*
SRS618815	MMETSP0799	*Cr-Ge-Ge2*	*Geminigera cryophila*	*Eukaryota*	*Cryptophyta*	*Geminigeraceae*

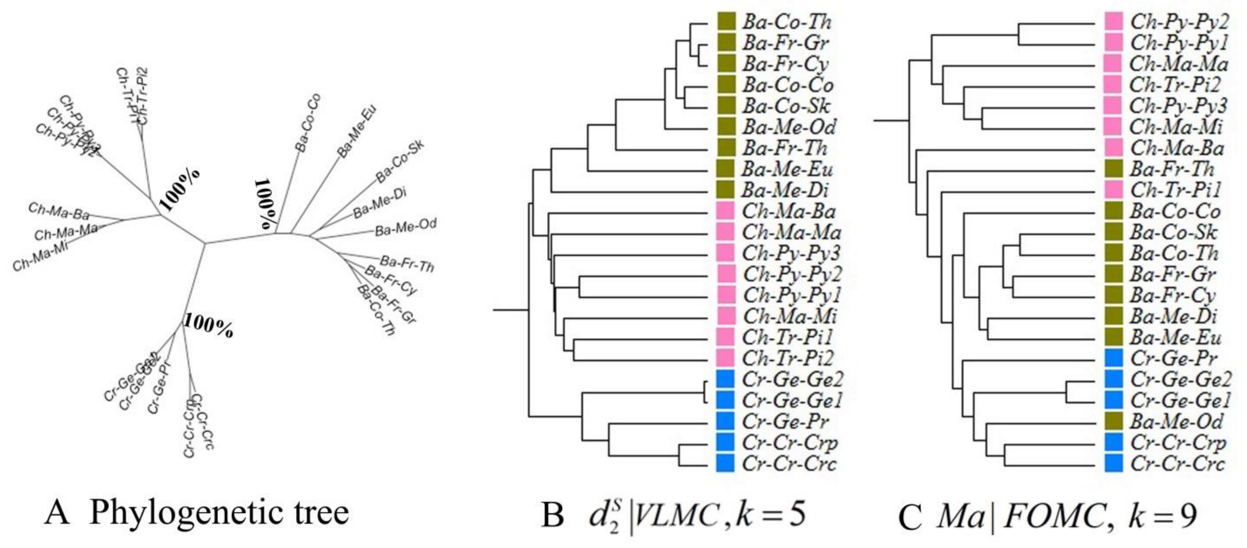

Figure S1. Reference tree and the derived clustering trees based on different models for 22 RNA-Seq data of microbial eukaryotes in experiment 2. (A) The phylogenetic tree of 22 RNA-Seq data built with MrBayes3. (B) The best clustering tree with VLMC. (C) The best clustering tree with FOMC and Lp-norm measures. * Samples are labeled as “phylum-class-organism”. For example, for sample Ba-Fr-Cy, Ba represents phylum

***Bacillariophyta*, Fr represents class *Fragilariophyceae*, and Cy represents the organism *Cyclophora tenuis*. Details about sample labels can be found in Table S2. Posterior probabilities with 100% support are shown at nodes in Figure S1 (A).**

Table S3 shows the Triple metric between the standard tree and the derived clustering using various dissimilarity measures and tuple length. The best clustering result with the smallest Triple metric of 469 is obtained from VLMC using dissimilarity measures d_2^S with tuple length k =5. Figure S1B shows the derived clustering using d_2^S based on VLMC and tuple length k =5. The samples are grouped according to phylum first and then class. The samples from the same phyla are clustered together very well. However, samples from the same class cannot be clearly grouped together. The smallest Triple metric of FOMC is 750, which was achieved by using Ma and k = 9. Figure S1C shows the corresponding clustering results, and even samples from the same phylum cannot be grouped together. The clustering result based on VLMC is obviously better than the result based on d_2^S or d_2^* under the FOMC model.

Table S3. Triples metric between the reference tree and the derived clustering tree using various dissimilarity measures and background sequence models of 22 RNA-Seq data of microbial eukaryotes in Experiment 2

k		2	3	4	5	6	7	8	9
VLMC	d_2^S	1114	697	656	***469***	645	647	791	800
	d_2^*	1091	739	630	564	643	726	799	878
FOMC	$d_2^S \mid M_0$	963	941	899	840	853	857	869	922
	$d_2^S \mid M_1$	1114	881	950	912	876	945	953	947
	$d_2^S \mid M_2$	NA	1103	944	874	979	933	909	798
	$d_2^S \mid M_3$	NA	NA	1028	855	910	934	932	811
	$d_2^* \mid M_0$	917	902	902	900	905	979	976	1010
	$d_2^* \mid M_1$	1091	895	951	903	917	975	1038	1046
	$d_2^* \mid M_2$	NA	973	890	709	1007	1017	1055	1037
	$d_2^* \mid M_3$	NA	NA	946	935	871	934	1055	1059
L_p -Norm and d_2 measures	d_2	905	923	930	931	954	952	932	982
	Ch	848	929	943	448	945	942	988	1045
	Eu	925	923	930	951	952	942	941	1015
	Ma	935	923	923	923	923	918	890	***750***

2. Comparison based on 88 global ocean metatranscriptomic samples in Experiment 3

Table S4. Description of datasets from 13 communities/projects used in experiment 3

<table>
<tr><th>Dataset ID & Name</th><th>Sequencing Platform</th><th>Database</th><th>Accession number in Database</th><th>Type and Number of Samples</th><th>Latitude</th><th>Longitude</th></tr>
<tr><td>1. Hawaii_Aug_DOM</td><td>454</td><td>CAMERA</td><td>CAM_P_0000715</td><td>7 MT (4 control &3 Amended)</td><td>23.18533333</td><td>-158.9393333</td></tr>
<tr><td>2. Hawaii_Aug_DSW</td><td>454</td><td>CAMERA</td><td>CAM_P_0000766</td><td>10 MT (5 control & 5 Amended)</td><td>23.18533333</td><td>-158.9393333</td></tr>
<tr><td>3. Hawaii_Nov_Day Night</td><td>454</td><td>CAMERA</td><td>CAM_PROJ_DayNight</td><td>2 MT</td><td>23.18533333</td><td>-158.9393333</td></tr>
<tr><td>4. Georgia</td><td>454</td><td>CAMERA</td><td>CAM_PROJ_CAM_P0000101</td><td>6 MT (2 control & 2 PUT & 2 SPD)</td><td>31.47744</td><td>-81.24176</td></tr>
<tr><td>5. Mexico</td><td>454</td><td>CAMERA</td><td>CAM_PROJ_DICE</td><td>4 MT (2 control & 2 Amended)</td><td>30.050233</td><td>-87.829467</td></tr>
<tr><td>6.Eastern Equa Atlan_Amazon</td><td>454</td><td>CAMERA</td><td>CAM_PROJ_AmazonRiverPlume</td><td>19 MT</td><td>12.28466667</td><td>56.1245</td></tr>
<tr><td>7. California_Deep sea</td><td>454</td><td>CAMERA</td><td>CAM_P_0000545</td><td>4 MT</td><td>27.506</td><td>-111.3469667</td></tr>
<tr><td>8. Norway</td><td>454</td><td>CAMERA</td><td>CAM_PROJ_PML</td><td>4 MT (2 control & 2 Amended)</td><td>60.269444</td><td>5.2222223</td></tr>
<tr><td>9. Hawaii_depth</td><td>454</td><td>CAMERA</td><td>CAM_PROJ_HOT</td><td>4 MT</td><td>23.18533333</td><td>-158.9393333</td></tr>
<tr><td>10. SWGE</td><td>454</td><td>CAMERA</td><td>CAM_PROJ_GeneExpression</td><td>14 MT</td><td colspan="2">None</td></tr>
<tr><td>11. WesternEnglish</td><td>454</td><td>CAMERA</td><td>CAM_PROJ_WesternChannelOM</td><td>8 MT & 8 MG</td><td>50.251667</td><td>-4.208889</td></tr>
<tr><td rowspan="4">12. NPSG</td><td>454</td><td>CAMERA</td><td>SRA007802.3/SRA007804.3/</td><td rowspan="2">4 MG62 replicates
&</td><td rowspan="4">23.18533333</td><td rowspan="4">-158.9393333</td></tr>
<tr><td>454</td><td>CAMERA</td><td>SRA007806.3/SRA000263/</td></tr>
<tr><td>454</td><td>CAMERA</td><td>SRA007801.5/SRA000262/</td><td rowspan="2">4 MT2 replicates</td></tr>
<tr><td>454</td><td>CAMERA</td><td>SRA007803.3/SRA007805.4</td></tr>
<tr><td>13. Mouse_Intestinal</td><td>Illumina</td><td>NCBI SRA</td><td>SRA051354</td><td>7 MT</td><td colspan="2">None</td></tr>
<tr><td colspan="7">*MT: Metatranscriptomic data; MG: Metagenomic data; PUT: Putrescine; SPD: Spermidine; DOM: Dissolved Organic Matter; DSW: Deep SeaWater. The 12 datasets are collected from CAMERA and NCBI SRA datasets. Some of them have corresponding datasets, others are submitted to datasets. The first column indicates the symbol name in this paper.</td></tr>
</table>

3. Comparison of gradient relationships based on metatranscriptomic samples from different iron-rich microbial mats in Experiment 5

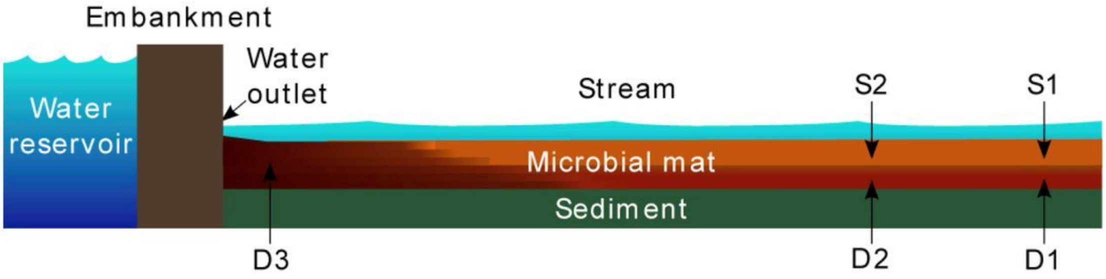

Figure S2. Sampling sites[2] of 14 metatranscriptomic samples in Experiment 5

Table S5. Reference information for iron-rich metatranscriptomic samples in experiment 5

Assay_Type	Run	Sample_Name	Depth	LibraryLayout	Organism_s	Platform
RNA-Seq	ERR435880	S1_11_sang_des_fees	1 cm below	SINGLE	microbial mat metagenome	LS454
RNA-Seq	ERR435899	S1_12_sang_des_fees	1 cm below	SINGLE	microbial mat metagenome	LS454
RNA-Seq	ERR435900	S1_13_sang_des_fees	1 cm below	SINGLE	microbial mat metagenome	LS454
RNA-Seq	ERR435901	S2_41_sang_des_fees	1 cm below	SINGLE	microbial mat metagenome	LS454
RNA-Seq	ERR435902	S2_42_sang_des_fees	1 cm below	SINGLE	microbial mat metagenome	LS454
RNA-Seq	ERR435903	S2_43_sang_des_fees	1 cm below	SINGLE	microbial mat metagenome	LS454
RNA-Seq	ERR435904	D1_21_sang_des_fees	7-9 cm below	SINGLE	microbial mat metagenome	LS454
RNA-Seq	ERR435905	D1_22_sang_des_fees	7-9 cm below	SINGLE	microbial mat metagenome	LS454
RNA-Seq	ERR435906	D1_23_sang_des_fees	7-9 cm below	SINGLE	microbial mat metagenome	LS454
RNA-Seq	ERR435907	D2_31_sang_des_fees	7-9 cm below	SINGLE	microbial mat metagenome	LS454
miRNA-Seq	ERR435908	D2_32_sang_des_fees	7-9 cm below	SINGLE	microbial mat metagenome	LS454
RNA-Seq	ERR435909	D2_33_sang_des_fees	7-9 cm below	SINGLE	microbial mat metagenome	LS454
RNA-Seq	ERR435910	D3_51_sang_des_fees	7-9 cm below	SINGLE	microbial mat metagenome	LS454
RNA-Seq	ERR435911	D3_52_sang_des_fees	7-9 cm below	SINGLE	microbial mat metagenome	LS454

4. PCA with different k values for VLMC and FOMC in Experiment 5

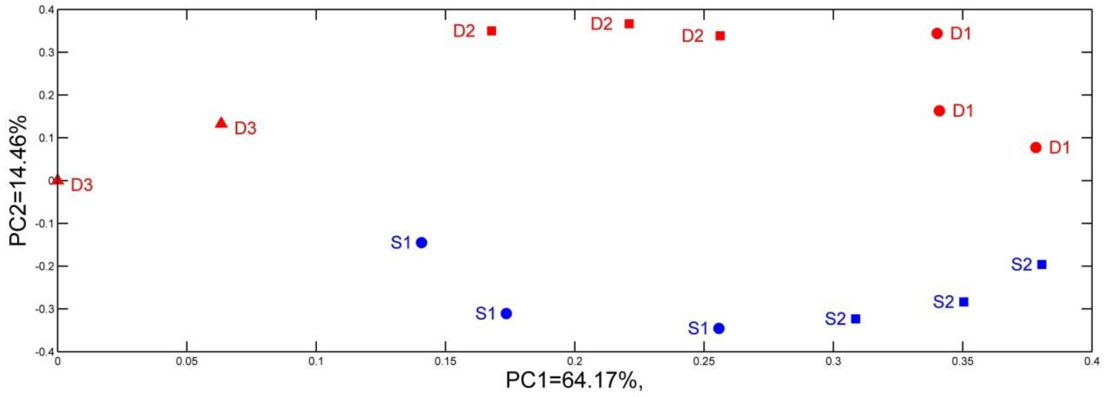

A: PCA ordination: $d_2^S|VLMC,k=7$

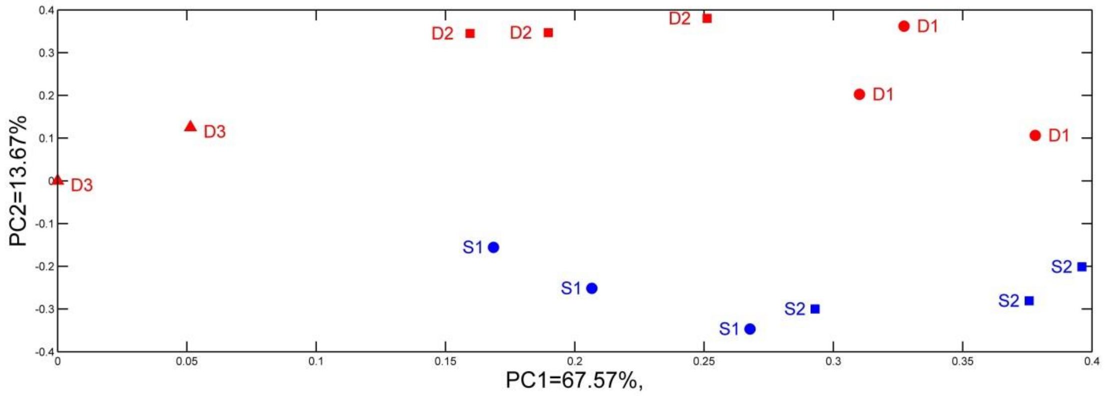

B: PCA ordination: $d_2^S|VLMC,k=9$

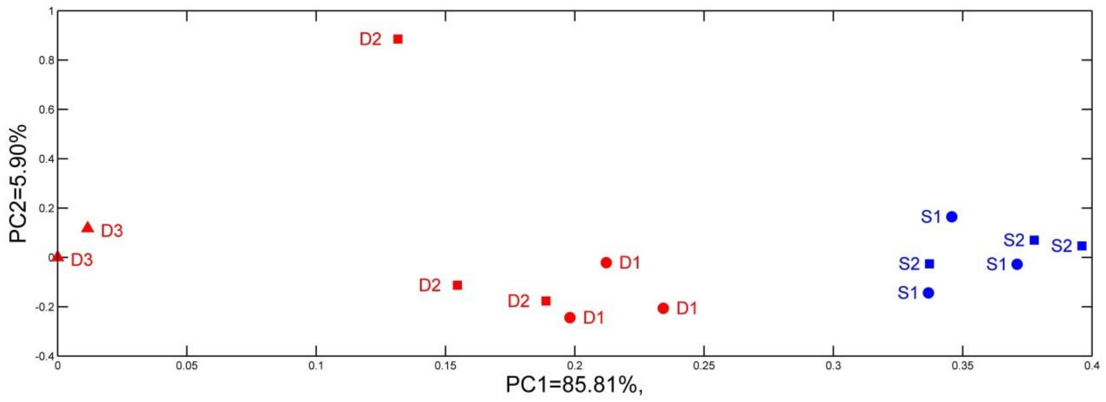

C: PCA ordination: $d_2^*|M_1,k=7$

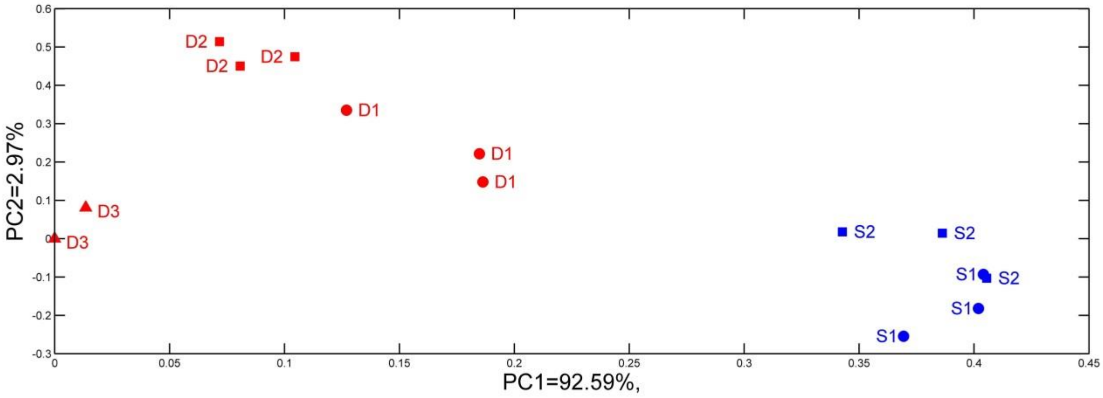

D: PCA ordination: $d_2^* | M_1, k = 9$

Figure S3. **PCA ordinates of samples with different k in Experiment 5**

5. Clustering profile of a real mammalian gut metagenomic dataset

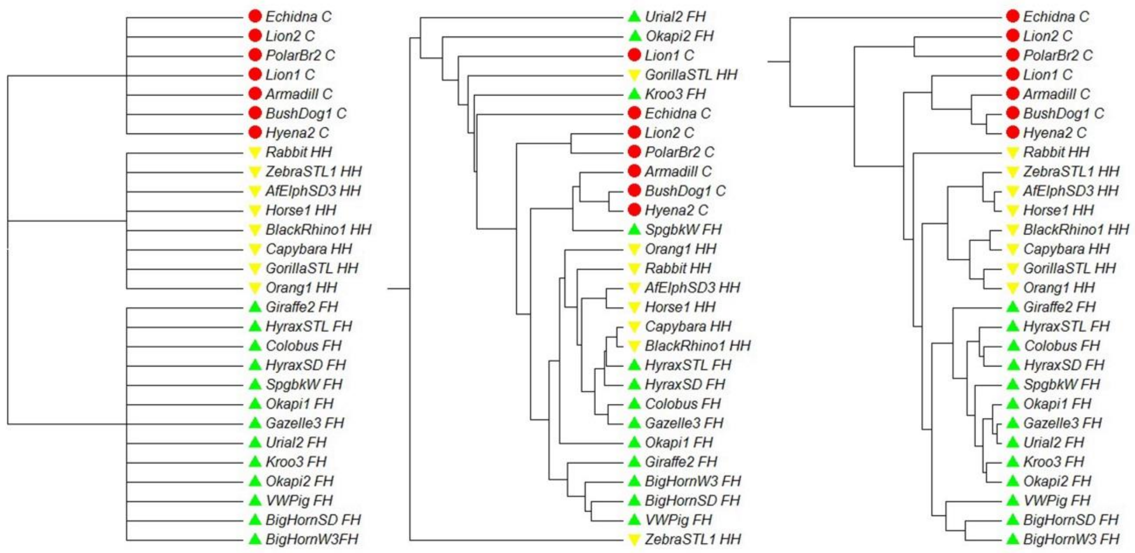

A Reference tree B $d_2^S|VLMC, k=6$ C $d_2^*|M_0, k=2$

Figure S4. Clustering results of the 28 mammalian gut samples: Green upward triangles denote foregut-fermenting herbivore; yellow downward triangles denote hindgut-fermenting herbivore; red discs denote simple-gut carnivore. (A) Reference tree of 28 mammalian gut samples. (B) Best clustering tree with VLMC and Lp-norm measures. (C) Best clustering tree with FOMC.

6. Comparison between FOMC model and VLMC model

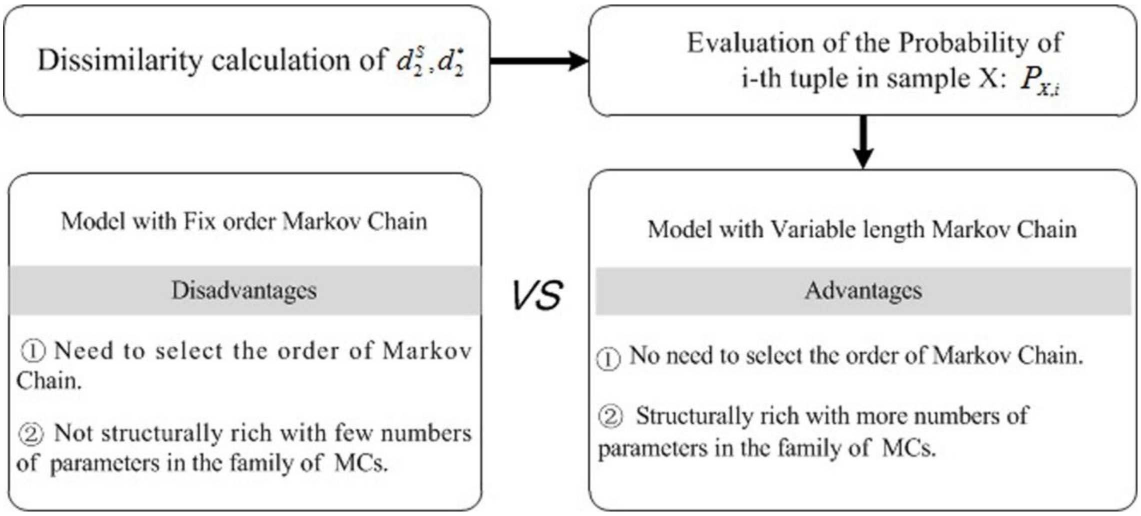

Figure S5. **FOMC model (left) compared to VLMC model (right)**

7. Comparison among Triple metric, Parsimony score and Symmetric difference scores

We made the comparison among Triple metric, Parsimony scores and Symmetric difference. It is found that Triple metric can measure the difference most accurately than other two difference metrics. The Symmetric difference is the number of nodes that are in one tree and not in the other one. Parsimony score for a tree is the sum of the smallest number of substitutions needed comparing with the reference tree. Compared with Parsimony score, Symmetric difference takes the order of hierarchical clustering into consideration.Triple metric can compare general leaf labelled rooted trees of arbitrary degree[3]. The algorithm enumerates all subsets of leaves of size three and counts how often the topologies induced by the three agree in the two trees.

We found that Triple metric can measure the distance between two rooted bifurcating trees more accurately than Symmetric difference. Take the Figure S6 as an example, Tree B and C has one and two misclassifications respectively. The Symmetric difference between Tree B/C and Tree A are both 8 and the Parsimony scores for Tree B/C are both 1, which cannot reflect the better performance of Tree B. And the distance measured by Triple metric between Tree B&A is 40 and Tree C&A is 55, which reflect the better performance of Tree B.

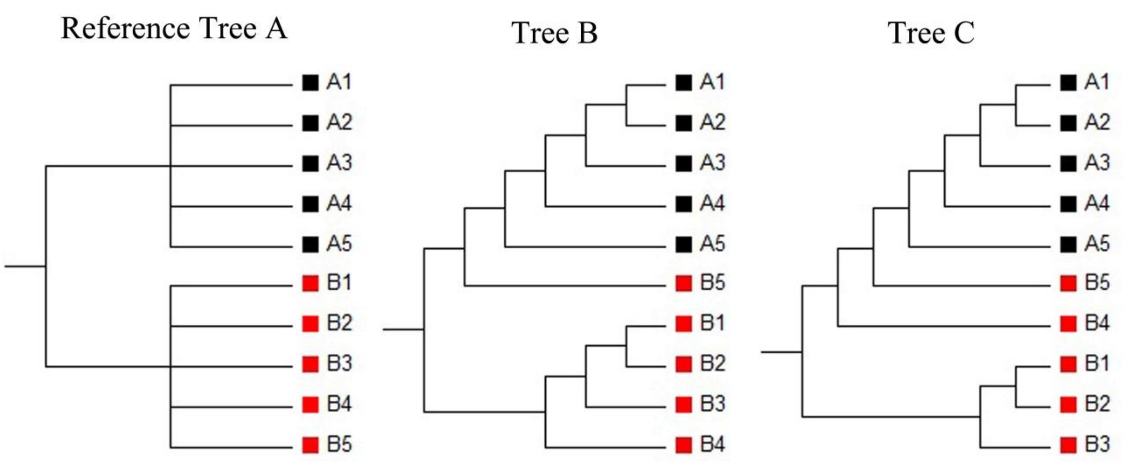

Figure S6. Example trees to compare Triple metric, Parsimony score and Symmetric difference scores

Furthermore, we listed the Triple metric, Parsimony scores and Symmetric difference scores table of Experiment 5 in Table S6 to further illustrate the consistence between the three indexes and the accuracy of Triple metric than Symmetric difference and Parsimony score. In Table S6, the smallest Triples metric is formatted in bold black text, the smallest Symmetric differences is in bold red font and the smallest Pasimony score is in bold purple font. It is shown that a group of trees with same scores for both Symmetric differences and pasimony scores, however, have more hierarchical and diverse rating scores for the Triples metric. Based on the above analysis, more-refined comparison can be implemented with the Triples metric when measuring the difference among trees and we adopted Triple metric as the index to compare the topological difference between reference and clustering trees in our study.

Table S6.Comparison between Triples metric(abbreviated as *tt*), Symmetric differences(abbreviated as *sd*) and Pasimony scores (abbreviated as *ps*)between the reference tree and the derived clustering tree using various dissimilarity measures and background sequence models to identify the gradient relationships of metatranscriptomic samples of microbial mats in Experiment 5.

	k		2	3	4	5	6	7	8	9
VLMC	d_2^S	*tt*	251	251	283	274	245	195	***76***	***76***
		sd	14	14	14	14	14	14	***10***	***10***
		ps	9	10	10	9	9	8	***5***	6
	d_2^*	*tt*	251	274	273	263	228	210	***76***	262
		sd	14	14	14	14	14	14	***10***	14
		ps	9	9	9	9	8	7	***5***	9
FOMC	$d_2^S\|M_0$	*tt*	218	189	189	202	202	178	201	189
		sd	14	14	14	14	14	***12***	14	14
		ps	8	8	8	8	7	***5***	6	6
	$d_2^S\|M_1$	*tt*	251	248	159	202	201	178	201	189
		sd	14	14	14	14	14	***12***	14	14
		ps	9	9	8	8	6	***5***	6	6
	$d_2^S\|M_2$	*tt*	NA	276	154	197	201	178	201	189

		sd	NA	14	***12***	14	14	***12***	14	14
		ps	NA	10	8	8	6	***5***	6	6
	$d_2^S \mid M_3$	*tt*	NA	NA	272	201	118	164	170	189
		sd	NA	NA	14	14	***12***	14	14	14
		ps	NA	NA	10	6	***5***	***5***	***5***	6
	$d_2^* \mid M_0$	*tt*	251	222	221	159	202	178	178	***148***
		sd	14	14	14	14	14	***12***	***12***	***12***
		ps	9	8	9	7	7	6	***5***	***5***
	$d_2^* \mid M_1$	*tt*	251	218	159	202	195	178	***148***	***148***
		sd	14	14	14	14	14	***12***	***12***	***12***
		ps	9	8	7	8	7	***5***	***5***	***5***
	$d_2^* \mid M_2$	*tt*	NA	274	154	154	195	178	***148***	***148***
		sd	NA	14	***12***	***12***	14	***12***	***12***	***12***
		ps	NA	9	8	7	6	***5***	***5***	***5***
	$d_2^* \mid M_3$	*tt*	NA	NA	268	201	195	***148***	***148***	***148***
		sd	NA	NA	14	14	14	***12***	***12***	***12***
		ps	NA	NA	9	7	6	***5***	***5***	***5***
L_p -Norm and $d_2$measures	d_2	*tt*	264	218	213	159	198	178	178	***148***
		sd	14	14	14	14	14	***12***	***12***	***12***
		ps	11	10	9	7	7	6	***5***	***5***
	Ch	*tt*	264	247	256	260	241	207	232	238
		sd	14	14	14	14	14	14	***12***	***12***
		ps	11	10	9	9	9	7	***5***	***5***
	Eu	*tt*	264	218	213	159	198	165	162	162
		sd	14	14	14	14	14	14	14	14
		ps	11	10	9	7	7	7	6	6
	Ma	*tt*	264	218	213	159	198	206	206	202
		sd	14	14	14	14	14	14	14	14
		ps	11	10	10	6	7	7	7	7

"M_i" indicates that the expected counts are calculated based on an i-th order Markov model for the background sequences. The p-values are estimated by comparing observed triples metric to the triples metric in 3000 randomly-joined trees: for all triples metric in table: p<0.001.

SUPPLEMENTARY REFERENCE

1 Duanmu, D. *et al.* Marine algae and land plants share conserved phytochrome signaling systems. *Proceedings of the National Academy of Sciences* **111**, 15827-15832 (2014).

2 Quaiser, A. *et al.* Unraveling the stratification of an iron-oxidizing microbial mat by metatranscriptomics. *PLoS One 9(7)* **e102561** (2014).

3 Schafer, M. & Crichlow, S. Antecedents of Groupthink A Quantitative Study. *Journal of Conflict Resolution* **40**, 415-435 (1996).
